# Comparative Genomics of *Pandoraea*, a Genus Enriched in Xenobiotic Biodegradation and Metabolism

**DOI:** 10.3389/fmicb.2019.02556

**Published:** 2019-11-06

**Authors:** Charlotte Peeters, Evelien De Canck, Margo Cnockaert, Evie De Brandt, Cindy Snauwaert, Bart Verheyde, Eliza Depoorter, Theodore Spilker, John J. LiPuma, Peter Vandamme

**Affiliations:** ^1^Laboratory of Microbiology, Department of Biochemistry and Microbiology, Faculty of Sciences, Ghent University, Ghent, Belgium; ^2^BCCM/LMG Bacteria Collection, Department of Biochemistry and Microbiology, Faculty of Sciences, Ghent University, Ghent, Belgium; ^3^Department of Pediatrics, University of Michigan Medical School, Ann Arbor, MI, United States

**Keywords:** *Pandoraea*, novel species, cystic fibrosis microbiology, comparative genomics, xenobiotics, biodegradation, opportunistic pathogens

## Abstract

Comparative analysis of partial *gyrB*, *recA*, and *gltB* gene sequences of 84 *Pandoraea* reference strains and field isolates revealed several clusters that included no taxonomic reference strains. The *gyrB*, *recA*, and *gltB* phylogenetic trees were used to select 27 strains for whole-genome sequence analysis and for a comparative genomics study that also included 41 publicly available *Pandoraea* genome sequences. The phylogenomic analyses included a Genome BLAST Distance Phylogeny approach to calculate pairwise digital DNA–DNA hybridization values and their confidence intervals, average nucleotide identity analyses using the OrthoANIu algorithm, and a whole-genome phylogeny reconstruction based on 107 single-copy core genes using bcgTree. These analyses, along with subsequent chemotaxonomic and traditional phenotypic analyses, revealed the presence of 17 novel *Pandoraea* species among the strains analyzed, and allowed the identification of several unclassified *Pandoraea* strains reported in the literature. The genus *Pandoraea* has an open pan genome that includes many orthogroups in the ‘Xenobiotics biodegradation and metabolism’ KEGG pathway, which likely explains the enrichment of these species in polluted soils and participation in the biodegradation of complex organic substances. We propose to formally classify the 17 novel *Pandoraea* species as *P. anapnoica* sp. nov. (type strain LMG 31117^T^ = CCUG 73385^T^), *P. anhela* sp. nov. (type strain LMG 31108^T^ = CCUG 73386^T^), *P. aquatica* sp. nov. (type strain LMG 31011^T^ = CCUG 73384^T^), *P. bronchicola* sp. nov. (type strain LMG 20603^T^ = ATCC BAA-110^T^), *P. capi* sp. nov. (type strain LMG 20602^T^ = ATCC BAA-109^T^), *P. captiosa* sp. nov. (type strain LMG 31118^T^ = CCUG 73387^T^), *P. cepalis* sp. nov. (type strain LMG 31106^T^ = CCUG 39680^T^), *P. commovens* sp. nov. (type strain LMG 31010^T^ = CCUG 73378^T^), *P. communis* sp. nov. (type strain LMG 31110^T^ = CCUG 73383^T^), *P. eparura* sp. nov. (type strain LMG 31012^T^ = CCUG 73380^T^), *P. horticolens* sp. nov. (type strain LMG 31112^T^ = CCUG 73379^T^), *P. iniqua* sp. nov. (type strain LMG 31009^T^ = CCUG 73377^T^), *P. morbifera* sp. nov. (type strain LMG 31116^T^ = CCUG 73389^T^), *P. nosoerga* sp. nov. (type strain LMG 31109^T^ = CCUG 73390^T^), *P. pneumonica* sp. nov. (type strain LMG 31114^T^ = CCUG 73388^T^), *P. soli* sp. nov. (type strain LMG 31014^T^ = CCUG 73382^T^), and *P. terrigena* sp. nov. (type strain LMG 31013^T^ = CCUG 73381^T^).

## Introduction

Members of the genus *Pandoraea* have emerged as rare opportunistic pathogens in persons with cystic fibrosis (Jørgensen et al., 2003; Johnson et al., 2004; Pimentel and MacLeod, 2008; Kokcha et al., 2013; Ambrose et al., 2016; Martina et al., 2017; See-Too et al., 2019) and several cases of chronic colonization and patient-to-patient transfer in this patient group have been reported (Jørgensen et al., 2003; Atkinson et al., 2006; Degand et al., 2015; Pugès et al., 2015; Ambrose et al., 2016; Dupont et al., 2017; Greninger et al., 2017). In addition to causing infection in cystic fibrosis patients, *Pandoraea* isolates have been recovered from blood and from samples from patients with chronic obstructive pulmonary disease or chronic granulomatous disease (Coenye et al., 2000; Daneshvar et al., 2001). Although the small number of patients involved and underlying diseases make it difficult to identify these bacteria as the cause of clinical deterioration (Martina et al., 2017; Green and Jones, 2018), one report described sepsis, multiple organ failure and death in a non-cystic fibrosis patient who underwent lung transplantation for sarcoidosis (Stryjewski et al., 2003).

Of the 11 validly named *Pandoraea* species, six (i.e., *Pandoraea apista*, *Pandoraea norimbergensis*, *Pandoraea pulmonicola*, *Pandoraea pnomenusa*, *Pandoraea sputorum*, and *Pandoraea fibrosis*) have been recovered from human clinical specimens (Coenye et al., 2000; See-Too et al., 2019), while *Pandoraea faecigallinarum*, *Pandoraea oxalativorans*, *Pandoraea terrae*, *Pandoraea thiooxydans*, and *Pandoraea vervacti* have been isolated from environmental samples (Anandham et al., 2010; Sahin et al., 2011; Jeong et al., 2016). An uncultivated endosymbiont of the trypanosomatid *Novymonas esmeraldas* represents an additional *Pandoraea* species which was provisionally named *Candidatus* Pandoraea novymonadis (Kostygov et al., 2017).

A growing number of reports demonstrate that soil and water represent the natural habitats of *Pandoraea* bacteria where they can be part of rhizosphere communities (Anandham et al., 2010; Jurelevicius et al., 2010; Peeters et al., 2016; Dong et al., 2018) and be involved in oxalate degradation (Jin et al., 2007; Sahin et al., 2011). The latter suggests they may be important contributors to soil formation, soil fertility and retention, and cycling of elements necessary for plant growth (Sahin, 2003). These free-living *Pandoraea* bacteria are often enriched in polluted soils and participate in the biodegradation of complex organic substances including lignin (Shi et al., 2013; Kumar et al., 2018b; Liu et al., 2019), biodiesel and petroleum by-products (de Paula et al., 2017; Sarkar et al., 2017; Tirado-Torres et al., 2017), *p*-xylene (Wang et al., 2015), δ-hexachlorocyclohexane (Pushiri et al., 2013), di-*n*-butyl phthalate (Yang et al., 2018), biphenyl, benzoate and naphthalene (Uhlik et al., 2012), and tetracycline (Wu et al., 2019) and β-lactam antibiotics (Crofts et al., 2017). A particularly well-documented *Pandoraea* strain, i.e., JB1^T^ (LMG 31106^T^), was isolated in the 1980s from garden soil (Parsons et al., 1988) and was able to use biphenyl, 2-, 3- and 4-chloro-biphenyl, *m*-toluate, *p*-toluate naphthalene, *m*-hydroxybenzoate and diphenylmethane (Springael et al., 1996). Although this strain also represented a separate novel *Pandoraea* species, it was not formally classified (Coenye et al., 2000) pending the availability of more than one strain representing the same novel species, a taxonomic practice that has been largely abandoned today.

The genome sequences of several strains with bioremediation potential have been reported, but a growing number of studies fail to provide species level identification of such strains (Pushiri et al., 2013; Chan et al., 2015; Kumar M. et al., 2016; Crofts et al., 2017; Liu et al., 2018; Wu et al., 2019). In addition, in our studies on the diversity and epidemiology of opportunistic pathogens in persons with cystic fibrosis, we isolated a considerable number of *Pandoraea* strains that represent novel species (unpublished data). The present study aimed to clarify the taxonomy and formally name these novel *Pandoraea* species, and to make reference cultures and whole-genome sequences of each of these versatile bacteria publicly available.

## Materials and Methods

### Bacterial Strains and Growth Conditions

Isolates representing novel *Pandoraea* species are listed in Table 1, along with their isolation source details. These strains were initially assigned to the genus *Pandoraea* on the basis of sequence analysis of 16S rRNA, *gyrB* or *recA* genes (data not shown). Well-characterized reference strains and recent field isolates identified in the present study as established *Pandoraea* species are listed in Supplementary Table S1. Strains were grown aerobically on Tryptone Soya Agar (Oxoid) and incubated at 28°C. Cultures were preserved in MicroBank^TM^ vials at −80°C.

**TABLE 1:** Isolates representing novel *Pandoraea* species.

**Strain**	**Other strains designations**	**Source**	**Depositor**
***Pandoraea anapnoica* sp. nov.**			
LMG 31117^T^	AU1288^T^, CCUG 73385^T^	CF patient (United States, 1999)	Own isolate
***Pandoraea anhela* sp. nov.**			
LMG 31108^T^	AU12140^T^, CCUG 73386^T^	CF patient (United States, 2006)	Own isolate
***Pandoraea aquatica* sp. nov.**			
LMG 31011^T^	CCUG 73384^T^	Pond water in greenhouse (Belgium, 2013)	Own isolate
***Pandoraea bronchicola* sp. nov.**			
LMG 20603^T^	CDC H652^T^, ATCC BAA-110^T^	CF sputum (United States, 1998)	CDC
R-10961	AU1775	CF patient (United States, 2000)	Own isolate
R-14318	AU2478	CF patient (United States, 2000)	Own isolate
R-52718	AU17726	CF patient (United States, 2009)	Own isolate
***Pandoraea capi* sp. nov.**			
LMG 20602^T^	CDC G9805^T^, ATCC BAA-109^T^	Non-CF sputum (United States, 1996)	CDC
R-15265	AU2777	CF patient (United States, 2001)	Own isolate
R-52714	AU12983	CF patient (United States, 2007)	Own isolate
***Pandoraea captiosa* sp. nov.**			
LMG 31118^T^	AU16660^T^, CCUG 73387^T^	CF patient (United States, 2008)	Own isolate
***Pandoraea cepalis* sp. nov.**			
LMG 31106^T^	JB1^T^, CCUG 39680^T^	Garden soil (Netherlands)	M. Mergeay
LMG 31107		Soil of house plant (Belgium, 2003)	Own isolate
R-51030		Pond water in greenhouse (Belgium, 2013)	Own isolate
***Pandoraea commovens* sp. nov.**			
LMG 31010^T^	CCUG 73378^T^	CF patient (Belgium, 2002)	C. De Boeck
LMG 24770	AI-S128	Plant root surface (India, 2002)	M. Madhaiyan
R-15662	AU3099	CF patient (United States)	Own isolate
***Pandoraea communis* sp. nov.**			
LMG 31110^T^	CCUG 73383^T^	CF patient (Belgium, 2012)	D. Pierard
LMG 31111		River water (Belgium, 2002)	Own isolate
R-17388		Maize rhizosphere soil (Belgium, 2002)	Own isolate
R-20591		River water (Belgium, 2002)	Own isolate
***Pandoraea eparura* sp. nov.**			
LMG 31012^T^	CCUG 73380^T^	Soil of house plant (Belgium, 2003)	Own isolate
***Pandoraea horticolens* sp. nov.**			
LMG 31112^T^	CCUG 73379^T^	Garden soil (Belgium, 2003)	Own isolate
***Pandoraea iniqua* sp. nov.**			
LMG 31009^T^	CCUG 73377^T^	Maize rhizosphere soil (Belgium, 2002)	Own isolate
LMG 31115	AU1290	CF patient (United States, 1999)	Own isolate
***Pandoraea morbifera* sp. nov.**			
LMG 31116^T^	AU12324^T^, CCUG 73389^T^	CF patient (United States, 2006)	Own isolate
R-54947	AU23671	CF patient (United States, 2011)	Own isolate
***Pandoraea nosoerga* sp. nov.**			
LMG 31109^T^	AU17017^T^, CCUG 73390^T^	CF patient (United States, 2008)	Own isolate
R-12863	AU2028	CF patient (United States, 2000)	Own isolate
R-13299	00BC460		P. Evans
R-15344	AU2347	CF patient (United States, 2000)	Own isolate
R-34565		CF patient (Australia, 2006)	M. Aravena-Roman
R-46874		CF patient (Belgium, 2011)	G. Ieven
R-47614		CF patient (Belgium, 2011)	H. Franckx
R-50065		CF patient (Belgium, 2012)	G. Ieven
R-50587		CF patient (Belgium, 2013)	C. De Boeck
R-52720	AU14034	CF patient (United States, 2007)	Own isolate
R-52722	AU18716	CF patient (United States, 2009)	Own isolate
***Pandoraea pneumonica* sp. nov.**			
LMG 31114^T^	AU18032^T^, CCUG 73388^T^	CF patient (United States, 2009)	Own isolate
***Pandoraea soli* sp. nov.**			
LMG 31014^T^	CCUG 73382^T^	Soil of house plant (Belgium, 2003)	Own isolate
***Pandoraea terrigena* sp. nov.**			
LMG 31013^T^	CCUG 73381^T^	Soil of house plant (Belgium, 2003)	Own isolate

*CF, cystic fibrosis. LMG, BCCM/LMG Bacteria Collection, Laboratory of Microbiology, Ghent University, Ghent, Belgium; CCUG, Culture Collection University of Gothenburg, Department of Clinical Bacteriology, Sahlgrenska University Hospital, Gothenburg, Sweden; CDC, Centers for Disease Control, United States Public Health Service, Atlanta, GA, United States.*

### DNA Preparation

DNA was extracted using an automated Maxwell^®^ DNA preparation instrument (Promega, United States). The final extract was treated with RNAse (2 mg/ml, 5 μL per 100 μL extract) and incubated at 37°C for 1 h. DNA quality was checked using 1% agarose gel electrophoresis and DNA quantification was performed using the QuantiFluor ONE dsDNA system and the Quantus fluorometer (Promega, United States). DNA was stored at −20°C prior to further analysis.

### Single Locus Sequence Analyses

Nearly complete 16S rRNA sequences were obtained as described previously (Peeters et al., 2013).

Partial *recA* gene sequences (663 bp) were amplified by PCR using forward primer 5′-AGG ACG ATT CAT GGA AGA WAG C-3′ and reverse primer 5′-GAC GCA CYG AYG MRT AGA ACT T-3′ (Spilker et al., 2009). Each 25 μl PCR reaction consisted of 1x PCR buffer (Qiagen), 1 U of Taq polymerase (Qiagen), 250 μM of each dNTP (Applied Biosystems), 1 × Q-solution (Qiagen), 1 μM of each primer and 2 μl of DNA (Peeters et al., 2013). PCR was performed using a Veriti 96 Well Thermal Cycler (Applied Biosystems). Initial denaturation for 2 min at 94°C was followed by 30 cycles of 30 s at 94°C, 45 s at 58°C and 1 min at 72°C, and a final elongation for 10 min at 72°C. Amplicons were purified using a NucleoFast 96 PCR clean-up kit (Macherey-Nagel). Sequencing primers (one per sequencing reaction) were the same as the amplification primers. Sequence analysis was performed with an Applied Biosystems 3130xl Genetic Analyzer and protocols of the manufacturer using the BigDye Terminator Cycle Sequencing Ready kit. Sequence assembly was performed using BioNumerics v7.6 (Applied Maths, Belgium).

Partial *gyrB* sequences (573 bp) were amplified by PCR using forward primer 5′-GAC AAY GGB CGY GGV RTB CC-3′ (this study) and reverse primer 5′-YTC GTT GWA RCT GTC GTT CCA CTG C-3′ (Spilker et al., 2009). The PCR protocol was the same as for *recA*, except that 2 μM of primer was used and an annealing temperature of 60°C. Sequencing primers (one per sequencing reaction) were 5′-ACG ACA AGC ACG ARC CSA AGC G-3′ (this study) and the same reverse primer as for amplification. Sequence analysis and assembly were performed as described above for the *recA* gene.

Partial *gltB* sequences were amplified by PCR using forward primer 5′-CTG CAT CAT GAT GCG CAA GTG-3′ (Spilker et al., 2009) and reverse primer 5′-GTT GCC ACG GAA RTC GTT GG-3′ (this study). The PCR protocol was the same as for *recA*, except that 0.4 μM of primer was used. Sequencing primers (one per sequencing reaction) were the same as the amplification primers. Sequence analysis and assembly were performed as described above for the *recA* gene.

Gene sequences of *recA*, *gyrB*, and *gltB* were aligned based on their amino acid sequences using Muscle (Edgar, 2004) in MEGA7 (Kumar S. et al., 2016). Phylogenetic trees were constructed using RAxML v8.2.11 (Stamatakis, 2014) with the GTRCAT substitution model and 1000 bootstrap analyses. Visualization and annotation of the phylogenetic trees was performed using iTOL (Letunic and Bork, 2016).

### Whole-Genome Sequencing

The genome sequences of 27 strains (Table 2 and Supplementary Table S2) were determined using the Illumina HiSeq4000 platform (PE150) at the Oxford Genomics Centre. Quality reports were created by FastQC. Reads were trimmed using Trimmomatic (Bolger et al., 2014) with the MAXINFO:50:0.8 and MINLEN:50 options. Genome size was estimated using kmc (Kokot et al., 2017) and reads were subsampled with seqtk^1^ to 80x coverage depth for assembly. Assembly was performed using SPAdes v3.12.0 (Bankevich et al., 2012) with error correction, default k-mer sizes (21, 33, 55, 77) and mismatch correction. Contigs were filtered on length (minimum 500 bp) and coverage (minimum 0.5x and maximum 8x overall coverage). Raw reads were mapped against the assemblies using bwa mem (Li, 2013) and contigs were polished using Pilon 1.22 (Walker et al., 2014) with default parameters. Quast (Gurevich et al., 2013) was used to create quality reports of the resulting assemblies. Annotation was performed using Prokka 1.12 (Seemann, 2014) with a genus-specific database based on publicly available genomes.

**TABLE 2:** Genomes included in the present study.

**Strain**	**Project**	**Contigs**	**Size(bp)**	**%GC**	**CDS**	**References**
*P. apista* DSM 16535^T^	PRJNA305052	2^*a*^	5,571,260	62.6	4,871	
*P. apista* LMG 18089	PRJEB30685	27	5,815,466	62.7	5,279	This study
*P. apista* AU2161	PRJNA284212	1^*a*^	5,574,863	62.7	4,655	Greninger et al., 2017
*P. apista* TF80G25	PRJNA281013	1^*a*^	5,609,637	62.6	4,691	Greninger et al., 2017
*P. apista* TF81F4	PRJNA271830	1^*a*^	5,582,097	62.6	4,676	Greninger et al., 2017
*P. apista* FDAARGOS_126	PRJNA231221	1^*a*^	5,326,503	62.7	4,621	
*P. apista* PA_200	PRJNA497126	82	5,677,857	62.5	4,969	
*P. apista* PA_201	PRJNA497126	69	5,680,846	62.5	4,957	
*P. apista* Pa13324	PRJNA507897	132	5,656,881	62.7	4,987	
*P. apista* Pa14367	PRJNA441551	97	5,596,190	62.8	4,932	
*P. apista* Pa14697	PRJNA507897	107	5,680,889	62.7	4,989	
*P. apista* Pa15518	PRJNA507897	106	5,631,909	62.7	4,924	
*P. apista* Pa15674	PRJNA507897	208	5,497,617	62.8	4,839	
*P. apista* Pa16226	PRJNA381452	23	5,724,490	62.7	4,998	
*P. apista* Pa16800	PRJNA507897	171	5,729,367	62.7	5,028	
*P. apista* Pa17292	PRJNA507897	113	5,621,546	62.7	4,909	
*P. apista* Pa18364	PRJNA507241	130	5,457,038	62.7	4,787	
*P. apista* Pa18384	PRJNA507897	136	5,678,267	62.7	4,914	
*P. apista* Pa18495	PRJNA507558	124	5,442,190	62.7	4,766	
*P. apista* Pa18531	PRJNA507897	143	5,525,740	62.7	4,817	
*P. faecigallinarum* DSM 23572^T^	PRJNA286722	3^*a*^	5,732,664	63.5	4,858	
*P. fibrosis* 6399^T^	PRJNA266749	70	5,574,251	62.9	4,356	Ee et al., 2015
*P. fibrosis* 7641	PRJNA266765	66	5,574,850	62.9	4,363	Ee et al., 2015
*P. fibrosis* LMG 31113	PRJEB30745	43	5,605,513	62.8	4,943	This study
*P. norimbergensis* DSM 11628^T^	PRJNA305058	1^*a*^	6,167,370	63.1	5,237	
*P. oxalativorans* DSM 23570^T^	PRJNA262701	5^*a*^	6,500,731	63.1	5,400	
*P. pnomenusa* DSM 16536^T^	PRJNA261997	1^*a*^	5,389,285	64.9	4,586	
*P. pnomenusa* LMG 31119	PRJEB30696	46	5,305,298	64.9	4,699	This study
*P. pnomenusa* 3kgm	PRJNA226227	1^*a*^	5,429,298	64.7	4,251	
*P. pnomenusa* E26	PRJNA229202	96	5,476,952	64.7	4,869	Chan et al., 2015
*P. pnomenusa* MCB032	PRJNA319140	4^*a*^	5,819,834	64.6	4,872	
*P. pnomenusa* RB38	PRJNA242373	1^*a*^	5,378,916	64.8	4,562	
*P. pnomenusa* RB-44	PRJNA230133	1^*a*^	5,385,946	64.9	4,646	
*P. pulmonicola* DSM 16583^T^	PRJNA270151	1^*a*^	5,867,621	64.3	4,868	
*P. sputorum* DSM 21091^T^	PRJNA262705	1^*a*^	5,742,997	62.8	4,884	
*P. sputorum* LMG 20601	PRJEB30706	62	6,264,179	62.7	5,539	This study
*P. sputorum* LMG 31120	PRJEB30707	17	5,956,418	62.7	5,301	This study
*P. sputorum* LMG 31121	PRJEB30708	113	6,453,978	62.8	5,652	This study
*P. terrae* LMG 30175^T^	PRJEB30813	81	6,176,823	62.8	5,575	This study
*P. thiooxydans* DSM 25325^T^	PRJNA285516	1^*a*^	4,464,186	63.2	3,999	
*P. thiooxydans* ATSB16	PRJNA309453	1^*a*^	4,464,185	63.2	4,388	
*P. vervacti* NS15^T^	PRJNA275368	2^*a*^	5,736,282	63.5	4,811	
*P. anapnoica* sp. nov. LMG 31117^T^	PRJEB30755	48	6,126,688	62.4	5,364	This study
*P. anhela* sp. nov. LMG 31108^T^	PRJEB30724	61	6,046,012	63.4	5,188	This study
*P. aquatica* sp. nov. LMG 31011^T^	PRJEB30756	17	5,958,127	62.9	5,238	This study
*P. bronchicola* sp. nov. LMG 20603^T^	PRJEB30725	34	5,351,123	63.0	4,753	This study
*P. capi* sp. nov. LMG 20602^T^	PRJEB30721	31	5,852,144	63.4	5,056	This study
*P. capi* sp. nov. ISTKB	PRJNA325244	115	6,367,971	63.2	5,356	Kumar M. et al., 2016
*P. captiosa* sp. nov. LMG 31118^T^	PRJEB30757	36	6,139,582	63.3	5,340	This study
*P. cepalis* sp. nov. LMG 31106^T^	PRJEB30715	56	5,274,229	63.7	4,730	This study
*P. cepalis* sp. nov. LMG 31107	PRJEB30716	32	5,159,566	63.5	4,626	This study
*P. cepalis* sp. nov. B-6	PRJNA169519	148	5,035,498	63.6	4,570	Liu et al., 2018
*P. commovens* sp. nov. LMG 31010^T^	PRJEB30753	26	6,036,949	62.6	5,308	This study
*P. communis* sp. nov. LMG 31110^T^	PRJEB30740	17	5,708,603	62.6	5,067	This study
*P. communis* sp. nov. LMG 31111	PRJEB30741	55	5,566,071	62.5	5,064	This study
*P. communis* sp. nov. SD6-2	PRJNA174277	37	5,772,015	62.5	5,148	Pushiri et al., 2013
*P. eparura* sp. nov. LMG 31012^T^	PRJEB30718	35	5,205,577	63.7	4,621	This study
*P. horticolens* sp. nov. LMG 31112^T^	PRJEB30744	68	6,008,490	62.3	5,378	This study
*P. iniqua* sp. nov. LMG 31009^T^	PRJEB30748	17	6,339,129	63.1	5,521	This study
*P. iniqua* sp. nov. LMG 31115	PRJEB30749	14	6,296,634	63.1	5,445	This study
*P. morbifera* sp. nov. LMG 31116^T^	PRJEB30750	47	5,233,298	64.7	4,676	This study
*P. nosoerga* sp. nov. LMG 31109^T^	PRJEB30729	41	4,862,114	66.1	4,266	This study
*P. pneumonica* sp. nov. LMG 31114^T^	PRJEB30747	12	5,845,078	62.5	5,202	This study
*P. soli* sp. nov. LMG 31014^T^	PRJEB30720	51	4,961,982	63.6	4,395	This study
*P. terrigena* sp. nov. LMG 31013^T^	PRJEB30719	35	5,356,606	63.5	4,878	This study
*Pandoraea* sp. PE-S2R-1	PRJNA385617	189	6,227,302	63.1	5,387	Crofts et al., 2017
*Pandoraea* sp. PE-S2T-3	PRJNA385617	37	6,176,158	63.2	5,310	Crofts et al., 2017
*Ca.* Pandoraea novymonadis E262	PRJNA369045	6	1,157,259	43.8	968	Kostygov et al., 2017

*^*a*^Status complete.*

### Publicly Available Genomes

All 41 publicly available (January 29, 2019) whole-genome sequences of *Pandoraea* bacteria were downloaded from the NCBI database (Table 2). *Burkholderia cenocepacia* J2315^T^ was used as an outgroup in the phylogenomic analyses. For strains B-6 (Liu et al., 2018), E26 (Chan et al., 2015), PE-S2R-1 and PE-S2T-3 (Crofts et al., 2017) no annotation was available and therefore annotation was performed using Prokka as described above.

### Phylogenomic Analyses

The GBDP approach was used to calculate pairwise digital DNA–DNA hybridization (dDDH) values and their confidence intervals (formula 2) using the Genome-to-Genome Distance Calculator (GGDC 2.1^2^) under recommended settings (Meier-Kolthoff et al., 2013). ANI values were calculated with the OrthoANIu algorithm (Yoon et al., 2017). Whole-genome phylogeny was assessed based on 107 single-copy core genes found in a majority of bacteria (Dupont et al., 2012) using bcgTree (Ankenbrand and Keller, 2016). Visualization and annotation of the phylogenetic tree was performed using iTOL (Letunic and Bork, 2016).

### Functional Genome Analyses

To enable a comparative genomic study, each protein-coding gene (CDS) in the 68 *Pandoraea* genomes (*n* = 331,123) was functionally classified using the COG (Galperin et al., 2015) and KEGG orthologies (Kanehisa and Goto, 2000; Kanehisa et al., 2017). COGs were assigned by a reversed position-specific BLAST (RPSBLAST v2.6.0+) with an *e*-value cut-off of 1E-3 against the NCBI conserved domain database (CDD v3.16) (Tange, 2011). KEGG orthology was inferred using the KEGG automated annotation server (KAAS) (Moriya et al., 2007). Based on COG and K numbers, each CDS was assigned to the respective COG category and KEGG hierarchy. In case multiple COG categories were defined for the same COG, the first category was considered as the primary category. Protein orthologous groups (orthogroups) were inferred using OrthoFinder v2.2.7 (Emms and Kelly, 2015) with default parameters. For each orthogroup, we mapped the genomes and species in which it was present, the specificity (core, multiple species, single species or single isolate), and COG and KEGG functional classification.

Data mapping, visualization and statistical analyses were performed using RStudio with R v3.5.2. Pearson’s chi-square analyses were used to test the association between different sets of categorical variables. When a significant relationship was found between two variables, we further examined the standardized Pearson residuals. Standardized Pearson residuals with high absolute values indicate a lack of fit of the null hypothesis of independence in each cell (Agresti, 2002) and thus indicate observed cell frequencies in the contingency table that are significantly higher or lower than expected based on coincidence.

### DNA Base Composition

The G + C content of all strains was calculated from their genome sequences using Quast (Gurevich et al., 2013).

### Biochemical Characterization

Biochemical characterization was performed as described previously (Draghi et al., 2014).

### Fatty Acid Methyl Ester Analysis

After a 24 h incubation period at 28°C on Tryptone Soya Agar (BD), a loopful of well-grown cells was harvested and fatty acid methyl esters were prepared, separated and identified using the Microbial Identification System (Microbial ID) as described previously (Vandamme et al., 1992).

## Results and Discussion

### Single Locus Sequence Analyses

**FIGURE 1:**
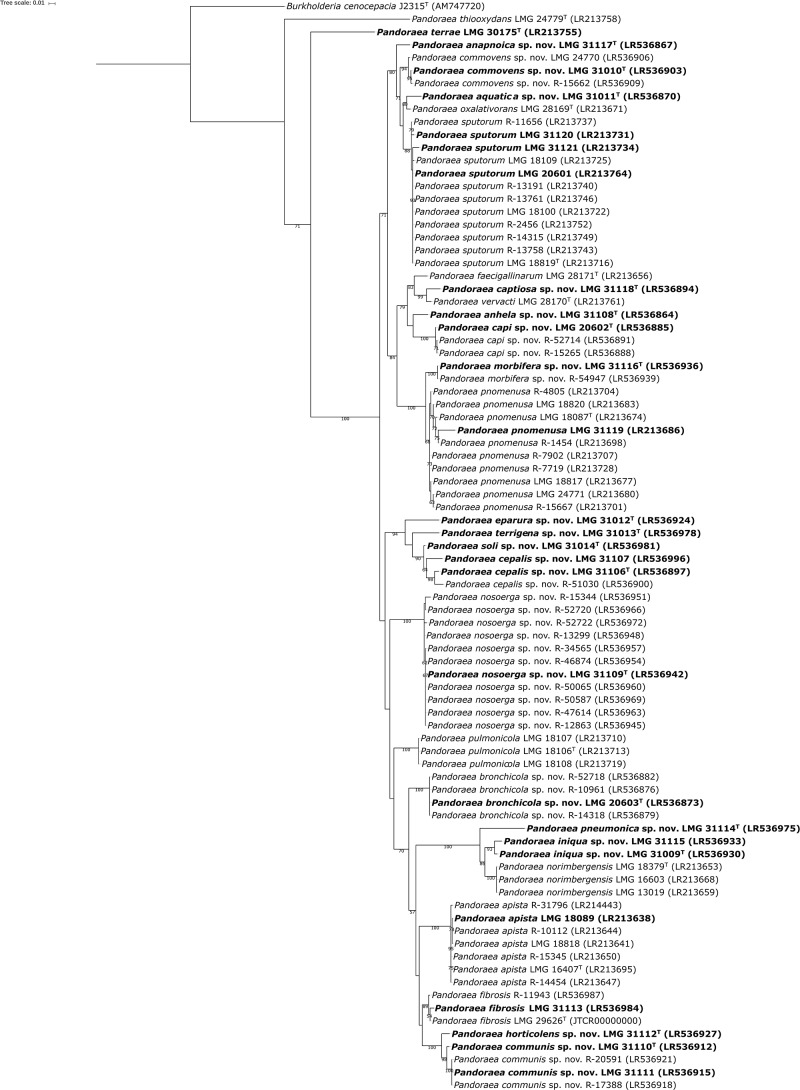
Phylogenetic tree based on partial *gyr*B sequences of all *Pandoraea* strains examined. Sequences (495–573 bp) were aligned based on their amino acid sequences and phylogeny was inferred using the Maximum Likelihood method and GTRCAT substitution model in RAxML. The percentage of replicate trees in which the associated taxa clustered together in the bootstrap test (1000 replicates) are shown next to the branches if greater than 50%. *Burkholderia cenocepacia* J2315^T^ was used as outgroup. The scale bar indicates the number of substitutions per site. Isolates selected for whole-genome sequencing are shown in bold character type.

The 16S rRNA gene sequences determined in the present study are publicly available through the GenBank/EMBL/DDBJ accession numbers listed in the species descriptions. Because the 16S rRNA sequences of *Pandoraea* species show high levels of similarity (Coenye et al., 2000; Daneshvar et al., 2001), *gyrB* gene sequence analysis has been introduced for species level identification of *Pandoraea* isolates (Coenye and LiPuma, 2002). To provide more robust phylogenetic analysis, partial sequences of the *gyrB* gene, and also of the *recA* and *gltB* genes were generated for a total of 84 *Pandoraea* reference strains and field isolates, and were used to construct phylogenetic trees (Figure 1 and Supplementary Figures S1, S2). The *gltB*, *gyrB* and *gltB* sequences determined in the present study are publicly available through the GenBank/EMBL/DDBJ accession numbers listed in Figure 1 and Supplementary Figures S1, S2 and in the species descriptions.

Overall, the three phylogenetic trees had comparable topologies, but while taxonomic reference strains of established *Pandoraea* species (Supplementary Table S1) and several groups of field isolates formed well-delineated clusters, others did not (Figure 1 and Supplementary Figures S1, S2). Each of these phylogenetic trees was therefore used to select a total of 27 isolates (shown in bold character type in Figure 1 and Supplementary Figures S1, S2) for whole-genome sequence analysis. These included 6 isolates that were tentatively assigned to established *Pandoraea* species using single locus sequence analyses, 20 isolates that clustered separately or whose assignment was equivocal, and *P. terrae* LMG 30175^T^, the sole *Pandoraea* type strain for which there was no publicly available whole-genome sequence at the time of writing.

### Genome Characteristics

The assembly of the Illumina HiSeq 150 bp paired end reads resulted in assemblies with 12–113 contigs and a total of 4.86–6.45 Mbp (Table 2 and Supplementary Table S2). The number of predicted CDS in the newly sequenced genomes ranged from 4,266 to 5,652 (Table 2). No clustered regularly interspaced short palindromic repeats (CRISPRs) were identified. The annotated assemblies of these 27 genomes were submitted to the European Nucleotide Archive and are publicly available through the GenBank/EMBL/DDBJ accession numbers listed in Table 2 and in the species descriptions. The G + C content of the newly sequenced strains, as calculated from their genome sequences, ranged from 62.3 to 66.1 mol% (Table 2). These values are similar those of other *Pandoraea* genomes, except for *Ca.* Pandoraea novymonadis that has a G + C content of 43.8% (Kostygov et al., 2017).

### Phylogenomic Analyses

**FIGURE 2:**
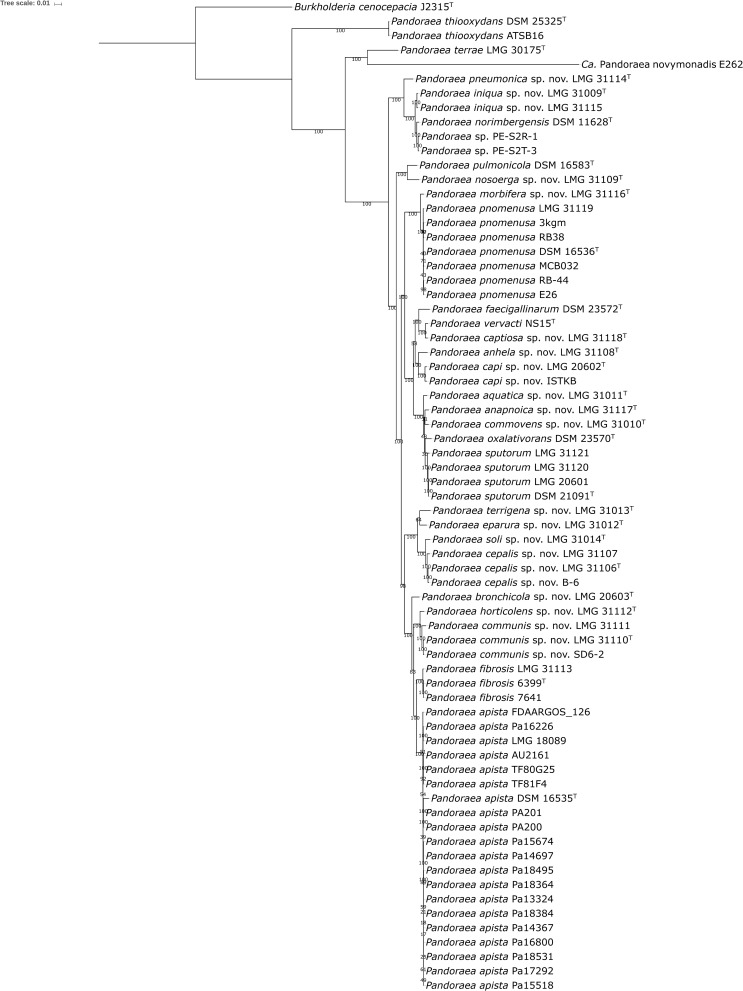
Phylogenetic tree based on 107 single-copy core genes. BcgTree was used to extract the nucleotide sequence of 107 single-copy core genes and to construct their phylogeny by partitioned maximum-likelihood analysis. The percentage of replicate trees in which the associated taxa clustered together in the bootstrap test (1000 replicates) are shown next to the branches. *Burkholderia cenocepacia* J2315^T^ was used as outgroup. Bar, 0.01 changes per nucleotide position.

The 27 genomes from the present study were compared to all 41 publicly available *Pandoraea* genomes (GenBank database, January 29, 2019), which included 6 unclassified *Pandoraea* strains (Pushiri et al., 2013; Chan et al., 2015; Crofts et al., 2017; Kumar et al., 2018a; Liu et al., 2018). Pairwise dDDH and ANI values among the 68 genome sequences were calculated and are listed in Supplementary Tables S3, S4, respectively. Species delineation based on the 70% dDDH (Meier-Kolthoff et al., 2013) and 95–96% ANI thresholds (Yoon et al., 2017) yielded 30 species, which included the 11 validly named species, *Ca.* Pandoraea novymonadis, a total of 17 novel species for which we propose the names shown in Table 1, and a novel species represented by strains PE-S2R-1 and PE-S2T-3 (Crofts et al., 2017) (see below). One of these novel species, i.e., *Pandoraea cepalis*, corresponds with *Pandoraea* genomospecies 1, which we reported earlier (Coenye et al., 2000). Two novel species, i.e., *Pandoraea capi* and *Pandoraea bronchicola*, correspond with *Pandoraea* genomospecies 3 and 4, respectively, reported by Daneshvar et al. (2001). Finally, the phylogenomic data (Figure 2 and Supplementary Tables S3, S4), but also each of the single locus sequence analyses, showed that *Pandoraea* genomospecies 2 LMG 20602 should be classified as *P. sputorum*, which contradicts earlier wet-lab DNA-DNA hybridization results (Daneshvar et al., 2001).

The use of dDDH and ANI threshold levels was generally straightforward, yet some pairs of strains showed values close to the generally applied taxonomic threshold levels (Supplementary Tables S3, S4) (Meier-Kolthoff et al., 2013; Yoon et al., 2017). The two strains classified as *P. capi* showed 96.4% ANI and 69.6% dDDH with a dDDH confidence interval of 66.6–72.5%, and these strains were therefore classified as the same species. Similarly, the three strains classified as *P. cepalis* showed 96.2–98.4% ANI, 68.4–86.0% dDDH, and the 70% dDDH threshold level was in the confidence interval; these strains were therefore classified as one species. *P. soli* LMG 31014^T^ showed 95.0–95.8% ANI and 60.7–65.0% dDDH toward *P. cepalis* strains, and the 70% dDDH threshold level was not part of the confidence interval, so this strain was classified as a separate species. Similarly, *P. horticolens* LMG 31112^T^ showed 95.0-95.3% ANI and 60.0-62.2% dDDH toward *P. communis*, and the 70% dDDH threshold level was not part of the confidence interval so this strain was also classified as a separate species.

The phylogenomic analyses also allowed us to identify 4 out of 6 unclassified *Pandoraea* strains for which genome sequences are publicly available: strain ISTKB (Kumar M. et al., 2016) was assigned to *P. capi*, strain B-6 (Liu et al., 2018) to *P. cepalis*, strain SD6-2 (Pushiri et al., 2013) to *P. communis*, and strain E26 (Chan et al., 2015) to *P. pnomenusa* (Figure 2 and Table 2). Finally, strains PE-S2R-1 and PE-S2T-3 (Crofts et al., 2017) formed a separate cluster, which represented yet another novel *Pandoraea* species that remains to be formally classified (Supplementary Tables S3, S4).

The phylogenomic tree based on 107 single-copy marker genes was well resolved and the clusters delineated by dDDH and ANI formed monophyletic groups with a high bootstrap support (Figure 2). The clades in the phylogenomic tree of the present study showed a branching order similar to a previously published tree based on 119 conserved proteins (Kostygov et al., 2017). The results of the phylogenomic analyses along with the clustering in the individual *recA*, *gyrB*, and *gltB* single locus sequence analyses (Figure 1 and Supplementary Figures S1, S2) were used to identify each of the 84 isolates included in the present study. *P. sputorum* strain LMG 31121 clustered with the remaining *P. sputorum* strains in the *gyrB* and *gltB* trees but grouped aberrantly in the *recA* tree. In addition, *P. cepalis* proved particularly difficult to identify through single locus sequence analysis as it exhibited more variation in each of the sequences examined (Figure 1 and Supplementary Figures S1, S2) than any other *Pandoraea* species.

### Phenotypic Characterization

The type strains of each of 11 established *Pandoraea* species and of 17 novel *Pandoraea* species reported in the present study were included in an extensive phenotypic characterization. Among *Pandoraea* species, *P. thiooxydans* not only occupies a separate phylogenetic position (Figures 1, 2 and Supplementary Figures S1, S2) but also has a distinctive phenotype (Table 3). While all other *Pandoraea* species show normal growth on general microbiological growth media (i.e., they generate colonies of 1–4 mm in diameter after 2 days of incubation at 37°C), *P. thiooxydans* LMG 24779^T^ requires prolonged incubation up to 7 days before the same colony size was obtained.

**TABLE 3:** Differential biochemical characteristics of all strains examined.

**Characteristic**	**1**	**2**	**3**	**4**	**5**	**6**	**7**	**8**	**9**	**10**	**11**	**12**	**13**	**14**	**15**	**16**	**17**	**18**	**19**	**20**	**21**	**22**	**23**	**24**	**25**	**26**	**27**	**28**
Growth at 45°C^*a*^	−	−	+	−	−	+	−	−	−	−	−	+	−	−	−	+	−	+	−	−	−	−	−	−	−	−	−	+
Growth at 5% NaCl	+	+	−	+	+	+	+	+	−	−	−	−	+	−	+	+	+	+	−	+	+	+	−	+	−	−	−	+
Growth at 6% NaCl	−	−	−	−	−	−	−	−	−	−	−	−	−	−	−	−	+	−	−	−	−	−	−	−	−	−	−	−
Growth on MacConkey agar	+	+	+	+	+	+	+	+	+	+	+	+	+	+	+	+	+	+	+	+	+	+	+	+	+	+	−	+
Catalase activity	+	+	+	+	+	+	+	−	+	+	+	+	+	+	+	+	+	+	+	+	+	+	+	+	+	+	+	+
Hydrolysis of tween 20	+	+	+	+	+	+	+	+	+	+	+	+	+	+	+	+	+	+	+	+	+	+	+	+	−	+	+	+
Hydrolysis of tween 80	−	−	−	+	−	−	−	−	−	−	−	−	−	−	+	−	−	−	−	−	−	−	+	−	−	−	−	−
Alkaline phosphatase	−	w	w	−	w	w	−	−	−	w	−	w	−	−	−	−	w	−	−	−	−	+	−	−	−	−	−	−
Acid phosphatase	+	+	+	+	w	w	+	w	+	+	w	+	+	w	+	+	+	w	w	+	w	+	−	−	−	w	+	+
C_4_ esterase activity	w	w	w	−	+	+	w	w	+	−	w	+	w	w	+	w	+	+	w	w	−	−	−	−	w	w	+	w
Naphtol-AS-BI-β-D-glucuronide	w	w	w	−	w	w	w	w	−	w	−	+	−	w	−	w	+	w	w	w	w	+	−	w	−	w	+	w
Nitrate reduction	−	−	−	−	−	−	−	+	+	−	+	−	−	−	−	+	−	−	−	−	+	−	+	−	−	+	+	−
Assimilation of D-glucose	−	−	−	−	−	−	−	−	−	−	−	−	−	−	−	−	+	−	−	−	−	−	−	−	−	−	w	−
Assimilation of D-gluconate	+	+	+	+	+	+	+	+	+	+	+	+	+	+	+	+	+	w	−	+	+	+	+	+	+	+	+	+
Assimilation of caprate	+	+	−	+	+	+	−	−	+	+	−	−	+	+	w	−	−	−	−	+	−	+	−	−	+	−	−	−
Assimilation of citrate	+	+	+	+	+	+	+	+	+	+	+	+	+	+	+	+	+	−	+	+	+	+	+	+	+	+	+	+
Assimilation of phenylacetate	+	+	+	+	+	+	+	+	+	+	+	+	+	+	+	+	+	−	+	+	+	+	+	+	+	+	+	+

*Species: 1, *P. anapnoica* LMG 31117^*T*^; 2, *P. anhela* LMG 31108^*T*^; 3, *P. apista* LMG 16407^*T*^; 4, *P. aquatica* LMG 31011^*T*^; 5, *P. bronchicola* LMG 20603^*T*^; 6, *P. capi* LMG 20602^*T*^; 7, *P. captiosa* LMG 31118^*T*^; 8, *P. cepalis* LMG 31106^*T*^; 9, *P. commovens* LMG 31010^*T*^*;* 10, *P. communis* LMG 31110^*T*^; 11, *P. eparura* LMG 31012^*T*^; 12, *P. faecigallinarum* LMG 28171^*T*^; 13, *P. fibrosis* LMG 29626^*T*^; 14, *P. horticolens* LMG 31112^*T*^; 15, *P. iniqua* LMG 31009^*T*^; 16, *P. morbifera* LMG 31116^*T*^; 17, *P. norimbergensis* LMG 18379^*T*^; 18, *P. nosoerga* LMG 31109^*T*^; 19, *P. oxalativorans* LMG 28169^*T*^; 20, *P. pneumonica* LMG 31114^*T*^; 21, *P. pnomenusa* LMG 18087^*T*^; 22, *P. pulmonicola* LMG 18106^*T*^; 23, *P. soli* LMG 31014^*T*^; 24, *P. sputorum* LMG 18819^*T*^; 25, *P. terrae* LMG 30175^*T*^*;* 26, *P. terrigena* LMG 31013^*T*^*;* 27, *P. thiooxydans* LMG 24779^*T*^; 28, *P. vervacti* LMG 28170^*T*^. +, present; −, absent; w, weak reaction. ^*a*^Growth characteristics were recorded after 4 days of incubation, except for *P. thiooxydans* LMG 24779^*T*^ for test results were recorded after 7 days of incubation.*

The following biochemical characteristics were shared by all *Pandoraea* strains investigated: growth at 15, 28, and 37°C, but not at 4°C; growth in the presence of 0–4% NaCl, but not in the presence of 6–10% NaCl; growth at pH 6, 7, and 8, but not at pH 4, 5, or 9. No anaerobic growth. Oxidase activity is present. No hydrolysis of starch or casein. No DNase activity. No denitrification. Assimilation of L-malate, but not L-arabinose, D-mannose, D-mannitol, *N*-acetylglucosamine, maltose or adipate. No fermentation of glucose. No indole production, esculin hydrolysis, arginine dihydrolase, urease or PNP-β-galactosidase activity, or liquefaction of gelatin. Leucine arylamidase activity is present, but no C_8_-ester-lipase, C_14_-lipase, valine or cystine arylamidase, trypsin, chymotrypsin, α-galactosidase, β-galactosidase, β-glucuronidase, α-glucosidase, β-glucosidase, *N*-acetyl-β-glucosaminidase, α-mannosidase or α-fucosidase activity.

An overview of biochemical characteristics useful for distinguishing the type strains of *Pandoraea* species is shown in Table 3.

The fatty acid profiles of all type strains are shown in Table 4. Both quantitative and qualitative differences were present. The predominant fatty acids in all strains investigated were C_16__:__0_, C_17__:__0_ cyclo, C_16__:__0_ 3-OH, C_18__:__1_ ω7c, C_19__:__0_ cyclo ω8c, summed feature 2 (comprising C_14__:__0_ 3-OH, C_16__:__1_ iso I, an unidentified fatty acid with equivalent chain length of 10.928, or C_12__:__0_ ALDE, or any combination of these fatty acids), or summed feature 3 (comprising C_16__:__1_ ω7c or C_15__:__0_ iso 2-OH or both).

**TABLE 4:** Fatty acid composition of all strains examined.

**Fatty acid**	**1**	**2**	**3**	**4**	**5**	**6**	**7**	**8**	**9**	**10**	**11**	**12**	**13**	**14**
C_12__:__0_	3.40	1.38	3.48	9.30	2.22	3.16	3.48	4.54	9.69	4.42	4.30	2.34	9.07	4.19
C_12__:__0_ 2-OH	Tr	1.09	1.99	1.09	1.90	1.30	1.24	ND	Tr	Tr	ND	Tr	1.61	Tr
C_14__:__0_	Tr	1.77	1.06	Tr	Tr	Tr	Tr	Tr	Tr	Tr	Tr	1.88	ND	Tr
C_14__:__0_ 2-OH	ND	1.01	ND	ND	ND	ND	ND	ND	ND	ND	ND	Tr	ND	ND
C_16__:__0_	18.95	20.54	20.21	18.38	23.45	16.09	15.29	15.29	17.76	18.82	15.47	15.77	19.98	17.45
C_16__:__0_ 2-OH	1.01	1.13	Tr	Tr	1.01	1.28	1.78	1.21	Tr	1.67	1.22	Tr	ND	1.56
C_16__:__0_ 3-OH	6.32	8.46	8.65	7.18	6.98	7.31	8.57	6.39	6.48	9.50	6.82	8.83	5.83	10.96
C_16__:__1_ 2-OH	1.29	Tr	4.27	1.08	1.45	2.04	1.99	3.99	Tr	1.76	3.47	5.00	ND	2.76
C_17__:__0_ cyclo	19.83	18.78	20.57	13.72	26.44	19.46	20.35	14.29	9.14	18.10	9.52	8.30	2.47	20.72
C_18__:__0_	Tr	ND	ND	Tr	ND	Tr	Tr	ND	Tr	Tr	Tr	Tr	Tr	ND
C_18__:__1_ 2-OH	3.39	3.35	3.91	3.07	2.49	5.18	5.94	2.71	2.17	3.15	3.54	5.86	ND	3.84
C_18__:__1_ ω7c	18.07	13.55	8.90	19.62	10.03	16.95	4.80	17.36	25.76	11.86	25.08	21.23	28.21	4.38
C_19__:__0_ cyclo ω8c	13.93	14.04	11.06	8.41	11.84	13.54	23.14	13.82	3.97	13.79	5.93	3.40	Tr	17.86
Summed feature 2^∗^	7.02	8.64	10.79	9.12	7.70	7.87	9.95	8.22	8.98	9.89	7.87	10.13	7.72	11.65
Summed feature 3^∗^	3.52	4.12	3.38	8.16	3.05	3.55	1.20	11.01	12.47	4.51	14.42	13.42	22.01	2.22

**Fatty acid**	**15**	**16**	**17**	**18**	**19**	**20**	**21**	**22**	**23**	**24**	**25**	**26**	**27**	**28**

C_12__:__0_	8.47	5.15	4.54	ND	3.93	4.18	2.03	3.02	4.31	2.86	5.59	4.35	3.97	4.18
C_12__:__0_ 2-OH	1.01	1.51	Tr	ND	Tr	Tr	1.72	1.98	ND	2.23	Tr	ND	ND	Tr
C_14__:__0_	Tr	1.19	Tr	1.71	Tr	Tr	Tr	Tr	Tr	Tr	Tr	Tr	Tr	Tr
C_14__:__0_ 2-OH	ND	ND	ND	8.27	ND	ND	Tr	ND	ND	ND	2.02	ND	ND	ND
C_16__:__0_	18.03	15.83	15.09	14.82	15.40	21.94	18.34	18.19	12.28	17.13	22.91	13.49	26.61	15.72
C_16__:__0_ 2-OH	ND	ND	1.06	3.66	1.26	1.61	1.33	1.79	1.67	1.14	ND	1.48	ND	1.16
C_16__:__0_ 3-OH	6.82	9.40	7.53	14.18	7.03	7.88	7.90	9.48	3.14	8.64	7.06	6.56	1.84	9.05
C_16__:__1_ 2-OH	Tr	1.07	2.41	2.20	4.93	1.35	2.78	2.19	5.96	Tr	2.00	4.84	Tr	3.51
C_17__:__0_ cyclo	14.43	4.84	22.11	15.45	11.45	22.81	16.32	16.55	8.49	14.59	8.24	11.33	19.60	19.36
C_18__:__0_	Tr	1.41	Tr	ND	Tr	Tr	Tr	Tr	Tr	ND	Tr	Tr	Tr	ND
C_18__:__1_ 2-OH	1.61	3.77	3.09	5.69	4.56	2.49	5.19	3.29	3.66	3.99	ND	3.85	Tr	6.58
C_18__:__1_ ω7c	21.55	30.31	12.94	1.25	20.47	8.85	17.84	7.05	16.75	21.18	18.83	23.91	12.42	9.54
C_19__:__0_ cyclo ω8c	7.75	5.58	14.19	13.03	8.57	14.85	10.16	20.19	3.33	11.56	5.31	5.65	3.12	17.50
Summed feature 2^∗^	8.63	9.75	10.50	18.27	8.30	9.07	8.49	11.07	12.00	9.87	9.45	8.15	12.29	9.59
Summed feature 3^∗^	9.10	9.26	3.58	ND	11.51	2.76	5.10	2.19	16.19	4.49	15.15	13.39	15.25	1.94

*Species: 1, *P. anapnoica* LMG 31117^*T*^; 2, *P. anhela* LMG 31108^*T*^; 3, *P. apista* LMG 16407^*T*^; 4, *P. aquatica* LMG 31011^*T*^; 5, *P. bronchicola* LMG 20603^*T*^; 6, *P. capi* LMG 20602^*T*^; 7, *P. captiosa* LMG 31118^*T*^; 8, *P. cepalis* LMG 31106^*T*^; 9, *P. commovens* LMG 31010^*T*^*;* 10, *P. communis* LMG 31110^*T*^; 11, *P. eparura* LMG 31012^*T*^; 12, *P. faecigallinarum* LMG 28171^*T*^; 13, *P. fibrosis* LMG 29626^*T*^; 14, *P. horticolens* LMG 31112^*T*^; 15, *P. iniqua* LMG 31009^*T*^; 16, *P. morbifera* LMG 31116^*T*^; 17, *P. norimbergensis* LMG 18379^*T*^; 18, *P. nosoerga* LMG 31109^*T*^; 19, *P. oxalativorans* LMG 28169^*T*^; 20, *P. pneumonica* LMG 31114^*T*^; 21, *P. pnomenusa* LMG 18087^*T*^; 22, *P. pulmonicola* LMG 18106^*T*^; 23, *P. soli* LMG 31014^*T*^; 24, *P. sputorum* LMG 18819^*T*^; 25, *P. terrae* LMG 30175^*T*^*;* 26, *P. terrigena* LMG 31013^*T*^*;* 27, *P. thiooxydans* LMG 24779^*T*^; 28, *P. vervacti* LMG 28170^*T*^. Those fatty acids for which the amount for all taxa was <1% are not included, therefore, the percentages may not add up to 100%. Tr, trace amount (<1%); ND, not detected. ^∗^Summed feature 2 comprises iso-C_16:1_I and/or C_14:0_3-OH; summed feature 3 comprises iso-C_15:0_2-OH and/or C_16:1_ω7c.*

### Functional Genome Analyses

**FIGURE 3:**
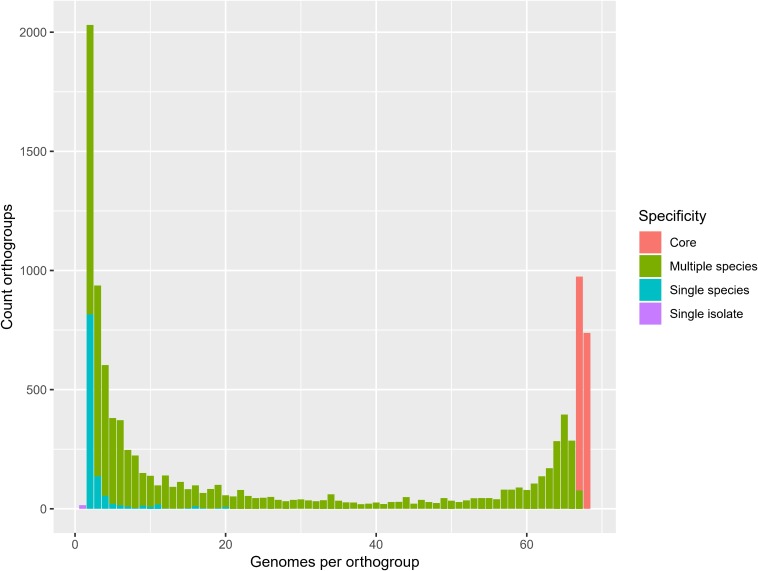
Genomes per orthogroup. Core, present in all genomes or all genomes except *Ca.* Pandoraea novymonadis.

The 68 *Pandoraea* genomes in the present study comprised 331,123 CDS, of which 273,692 (83%) and 128,054 (39%) could be assigned to the COG and KEGG orthologies, respectively (Supplementary Table S5). Orthologous genes were identified to determine the conserved genome content of the genus *Pandoraea*. Ortholog analysis revealed 10,783 orthogroups (325,879 CDS) in total, of which 738 (51,633 CDS) were present in all genomes, 897 (62,692 CDS) were present in all genomes except *Ca.* Pandoraea novymonadis, 8,003 (207,937 CDS) were present in multiple species, 1,130 (3,581 CDS) were species-specific and 15 (36 CDS) were isolate-specific (Figure 3). For further analyses, the core orthogroups were defined as those present in all genomes or all genomes except *Ca.* Pandoraea novymonadis (*n* = 1,635). COG and KEGG could be assigned to 7,243 (67%) and 3,655 (34%) of a total of 10,783 orthogroups (Supplementary Table S6). A previous pan genome analysis of 36 *Pandoraea* genomes by Wu et al. (2019) revealed a core genome of 1,903 CDS. As shown by these authors, the pan genome of *Pandoraea* is open (Wu et al., 2019) and the number of core genes decreases with an increasing number of genomes analyzed.

**FIGURE 4:**
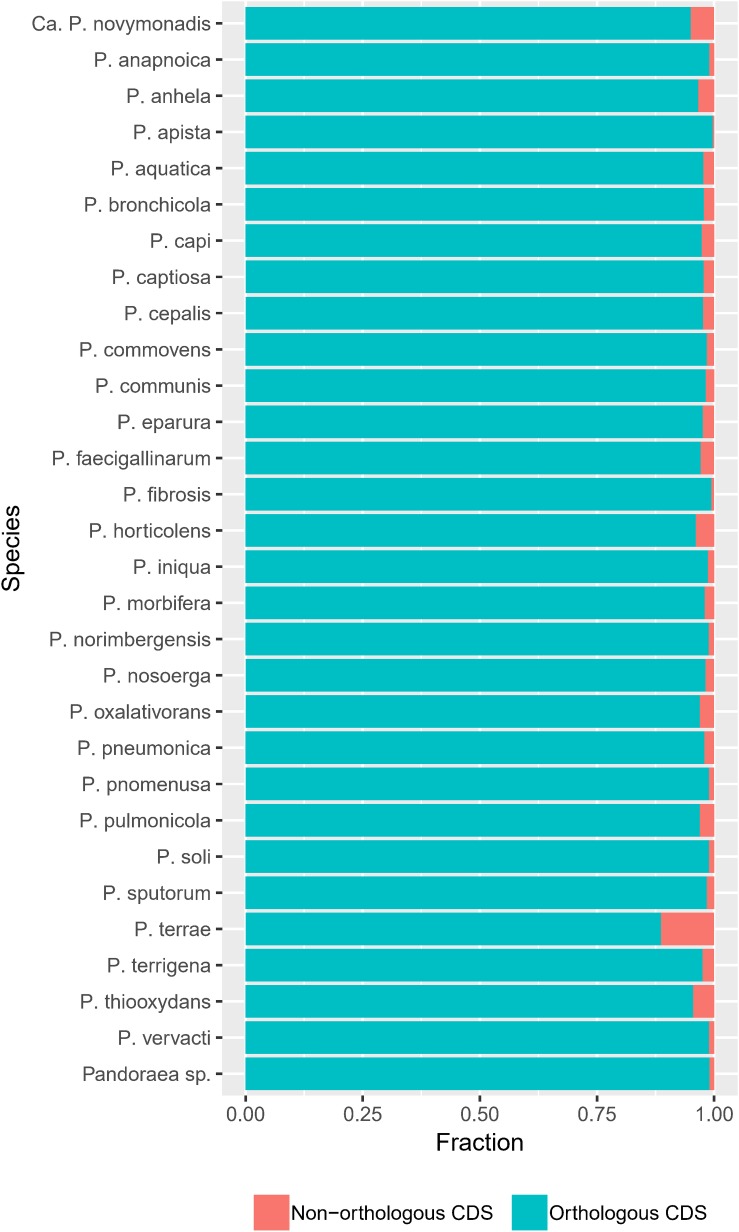
The frequency of orthologous versus non-orthologous CDS varies among species. Bar plots show the number of orthologous and non-orthologous CDS per species [*X*^2^(29) = 5863, *p* < 0.001].

**FIGURE 5:**
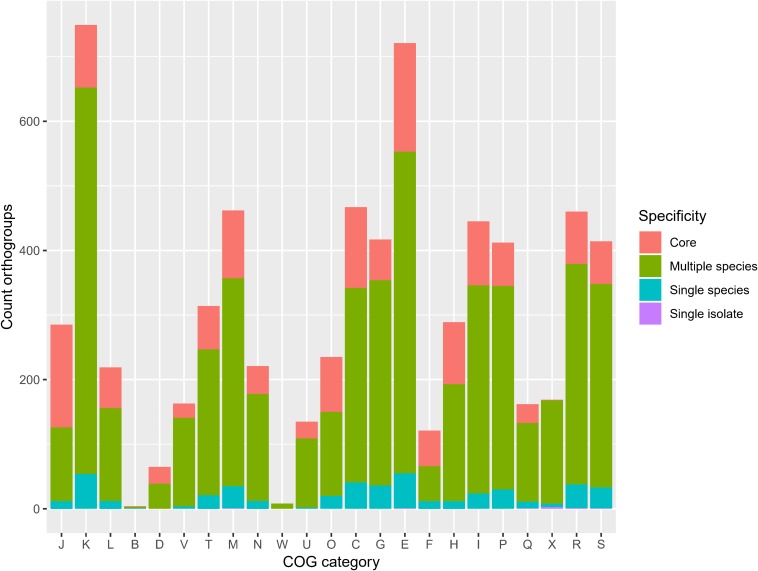
Orthogroup specificity varies among COG categories. Bar plot shows the number of orthogroups and their specificity per COG category [*X*^2^(66) = 522, *p* < 0.001]. J, translation, ribosomal structure and biogenesis; K, transcription; L, replication, recombination and repair; B, chromatin structure and dynamics; D, cell cycle control, cell division, chromosome partitioning; V, defense mechanisms; T, signal transduction mechanisms; M, cell wall/membrane/envelope biogenesis; N, cell motility; W, extracellular structures; U, intracellular trafficking, secretion, and vesicular transport; O, posttranslational modification, protein turnover, chaperones; X, mobilome: prophages, transposons; C, energy production and conversion; G, carbohydrate transport and metabolism; E, amino acid transport and metabolism; F, nucleotide transport and metabolism; H, coenzyme transport and metabolism; I, lipid transport and metabolism; P, inorganic ion transport and metabolism; Q, secondary metabolites biosynthesis, transport and catabolism; R, general function prediction only; S, function unknown.

**FIGURE 6:**
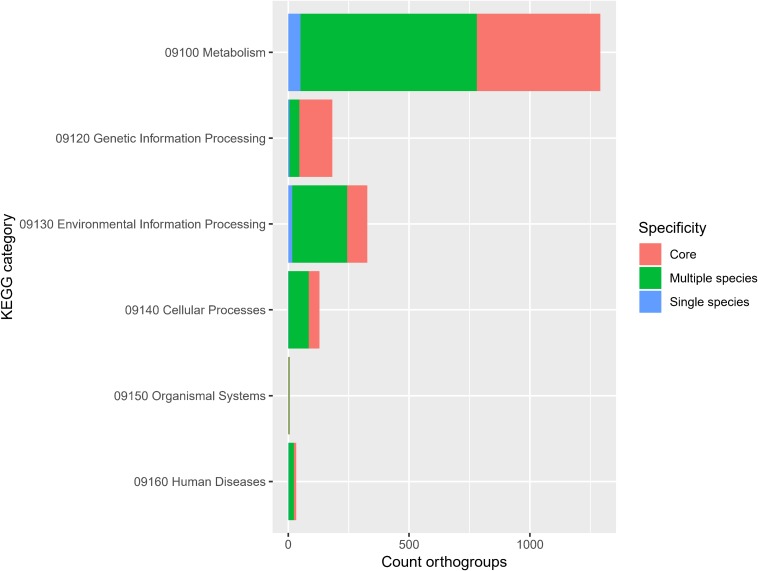
Orthogroup specificity varies among KEGG categories. Bar plot shows the number of orthogroups and their specificity per KEGG category [*X*^2^(10) = 130, *p* < 0.001].

The frequency of orthologous versus non-orthologous CDS varied significantly per isolate [*X*^2^(67) = 7423, *p* < 0.001] and species [*X*^2^(29) = 5863, *p* < 0.001]. The number of non-orthologous CDS per genome ranged from 0 to 632, with *P. terrae* LMG 30175^T^ showing the highest percentage of non-orthologous CDS (Figure 4 and Supplementary Table S7). To identify biological functions that were over- or underrepresented in the core genome, we looked at the COG and KEGG functional classification of the orthogroups versus their specificity (core, multiple species, single species or single isolate). The specificity of the orthogroups varied significantly among the COG categories [*X*^2^(66) = 522, *p* < 0.001] and highest levels of the KEGG pathways [*X*^2^(10) = 130, *p* < 0.001]. The core orthogroups were significantly enriched in the COG categories Translation, ribosomal structure and biogenesis (J), Posttranslational modification, protein turnover, chaperones (O), Nucleotide transport and metabolism (F) and Coenzyme transport and metabolism (H) (Figure 5 and Supplementary Table S8) and in the KEGG pathway Genetic Information Processing (09120) (Figure 6 and Supplementary Table S9).

**FIGURE 7:**
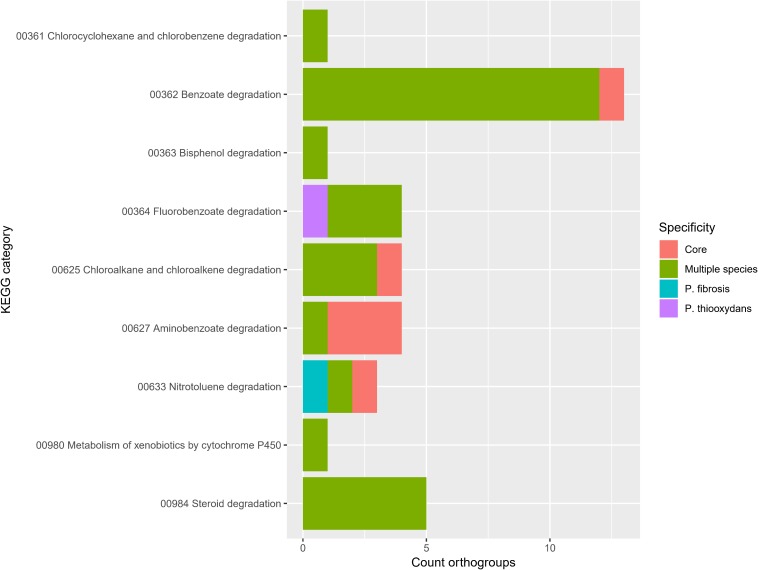
Orthogroup specificity in KEGG pathway Xenobiotics biodegradation and metabolism. Bar plot shows the number of orthogroups and their specificity.

Because many *Pandoraea* strains participate in the biodegradation of recalcitrant xenobiotics (Uhlik et al., 2012; Pushiri et al., 2013; Shi et al., 2013; Wang et al., 2015; Crofts et al., 2017; de Paula et al., 2017; Sarkar et al., 2017; Tirado-Torres et al., 2017; Kumar et al., 2018b; Yang et al., 2018; Liu et al., 2019; Wu et al., 2019), we specifically looked at the orthogroups in the KEGG pathway Xenobiotics biodegradation and metabolism (Figure 7). Most orthogroups in this pathway were present in multiple species (*n* = 28) and some were even present in the core *Pandoraea* genome (*n* = 6). This confirmed the potential of *Pandoraea* for degrading xenobiotics. In particular, the widespread capacity to utilize benzoate derivatives (Figure 7, pathways 362, 364, 627, and 633) explains why several strains have the potential to degrade lignin (Shi et al., 2013; Kumar et al., 2018a; Liu et al., 2019) and other aromatic compounds (Springael et al., 1996; Uhlik et al., 2012; Wang et al., 2015). Finally, *P. fibrosis* and *P. thiooxydans* showed a unique capacity to degrade specific compounds (Figure 7). *P. fibrosis* was only recently described and named after its origin from a cystic fibrosis patient (See-Too et al., 2019) but its unique capacity to degrade nitrotoluene derivatives is yet another example of the versatility in one *Pandoraea* species.

## Conclusion

The present study extends the number of formally named *Pandoraea* species considerably and makes reference cultures and their whole-genome sequences publicly available. The genus *Pandoraea* further emerges as a group of environmental bacteria with strong biodegradation capacities and as opportunistic human pathogens, especially in persons with cystic fibrosis. Within this genus, *P. thiooxydans* and *P. terrae* and *Candidatus* P. novymonadis cluster outside the main *Pandoraea* lineage. The aberrant phylogenomic position of the former is further supported by a distinctive phenotype. The classification of these bacteria within this monophyletic genus could therefore be questioned.

Taking into account the source and identification of strains ISTKB (a rhizospheric soil isolate, Kumar M. et al., 2016) and B-6 (an eroded bamboo slip isolate, Liu et al., 2018), and, to be as comprehensive as possible, also some additional unpublished own data (JL and PV), the novel species *P. aquatica*, *P. capi*, *P. cepalis*, *P. commovens*, *P. communis*, and *P. iniqua*, but also the established species *P. faecigallinarum*, *P. norimbergensis*, *P. pnomenusa*, and *P. fibrosis*, have all been isolated from both human clinical and environmental sources. Thus far, the novel species *P. anapnoica*, *P. anhela*, *P. bronchicola*, *P. captiosa*, *P. morbifera*, *P. nosoerga*, and *P. pneumonica*, but also the established species *P. apista*, *P. pulmonicola*, and *P. sputorum*, have all been isolated from human clinical sources only; while the novel species *P. eparura, P. horticolens, P. soli* and *P. terrigena*, and the established species *P. oxalativorans*, *P. terrae*, *P. thiooxydans*, and *P. vervacti* have thus far been isolated from environmental samples only.

The present study provides genomic, chemotaxonomic and phenotypic data that enable a formal proposal of 17 novel *Pandoraea* species as outlined below. By making reference cultures and whole-genome sequences of each of these versatile bacteria publicly available, we aim to contribute to future knowledge about the metabolic versatility and pathogenicity of these organisms.

### Description of *Pandoraea anapnoica* sp. nov.

*Pandoraea anapnoica* sp. nov. (a.na.pnoi’ca. Gr. masc. adj. *anapnoikos*, affecting respiration; N.L. fem. adj. *anapnoica* affecting respiration).

The phenotypic description is as presented above and in Table 3.

Isolated from human clinical samples in the United States.

The type strain is LMG 31117^T^ (=CCUG 73385^T^) and was isolated from a cystic fibrosis specimen in the United States in 1999. Its G + C content is 62.4 mol% (calculated based on its genome sequence). The 16S rRNA, *gltB*, *gyrB*, *recA* and whole-genome sequence of LMG 31117^T^ are publicly available through the accession numbers LR536847, LR536866–LR536868, and CABPSP010000000, respectively.

### Description of *Pandoraea anhela* sp. nov.

*Pandoraea anhela* sp. nov. (an.he’la. L. fem. adj. *anhela* breath-taking).

The phenotypic description is as presented above and in Table 3.

Isolated from human clinical samples in the United States.

The type strain is LMG 31108^T^ (=CCUG 73386^T^) and was isolated from a cystic fibrosis specimen in the United States in 2006. Its G + C content is 63.4 mol% (calculated based on its genome sequence). The 16S rRNA, *gltB*, *gyrB*, *recA* and whole-genome sequence of LMG 31108^T^ are publicly available through the accession numbers LR536848, LR536863-LR536865 and CABPSB010000000, respectively.

### Description of *Pandoraea aquatica* sp. nov.

*Pandoraea aquatica* sp. nov. (a.qua’ti.ca. L. fem. adj. *aquatica* aquatic).

The phenotypic description is as presented above and in Table 3.

Isolated from human clinical samples in the United States and from pond water in Belgium.

The type strain is LMG 31011^T^ (=CCUG 73384^T^) and was isolated from pond water in a greenhouse in Belgium in 2013. Its G + C content is 62.9 mol% (calculated based on its genome sequence). The 16S rRNA, *gltB*, *gyrB*, *recA* and whole-genome sequence of LMG 31011^T^ are publicly available through the accession numbers LR536849, LR536869–LR536871, and CABPSN010000000, respectively.

### Description of *Pandoraea bronchicola* sp. nov.

*Pandoraea bronchicola* sp. nov. (bron.chi’co.la. L. neut. pl. n. *bronchia*, the bronchial tubes; L. suff. -*cola* [from L. n. *incola*] a dweller, inhabitant; N.L. fem. n. *bronchicola* a dweller of bronchi, coming from the bronchi).

The phenotypic description is as presented above and in Table 3.

Isolated from human clinical samples in the United States.

The type strain is LMG 20603^T^ (= ATCC BAA-110^T^ = CDC H652^T^) and was isolated from cystic fibrosis sputum in the United States in 1998. Its G + C content is 63.0 mol% (calculated based on its genome sequence). The 16S rRNA, *gltB*, *gyrB*, *recA* and whole-genome sequence of LMG 20603^T^ are publicly available through the accession numbers LR536994, LR536872–LR536874, and CABPST010000000, respectively.

### Description of *Pandoraea capi* sp. nov.

*Pandoraea capi* sp. nov. (ca’pi. Gr. masc. n. *kapos*, breath; N.L. gen. n. *capi*, referring to the lung as niche of these bacteria).

The phenotypic description is as presented above and in Table 3.

Isolated from human clinical samples in the United States and from rhizospheric soil in India.

The type strain is LMG 20602^T^ (=ATCC BAA-109^T^ = CDC G9805^T^) and was isolated from sputum of a non-cystic fibrosis patient in the United States in 1996. Its G + C content is 63.4 mol% (calculated based on its genome sequence). The 16S rRNA, *gltB*, *gyrB*, *recA* and whole-genome sequence of LMG 20602^T^ are publicly available through the accession numbers LR536850, LR536884–LR536886, and CABPRV010000000, respectively.

### Description of *Pandoraea captiosa* sp. nov.

*Pandoraea captiosa* sp. nov. (cap.ti.o’sa. L. fem. adj. *captiosa*, harmful, disadvantageous).

The phenotypic description is as presented above and in Table 3.

Isolated from human clinical samples in the United States.

The type strain is LMG 31118^T^ (=CCUG 73387^T^) and was isolated from a cystic fibrosis specimen in the United States in 2008. Its G + C content is 63.3 mol% (calculated based on its genome sequence). The 16S rRNA, *gltB*, *gyrB*, *recA* and whole-genome sequence of LMG 31118^T^ are publicly available through the accession numbers LR536851, LR536893–LR536895, and CABPSQ010000000, respectively.

### Description of *Pandoraea cepalis* sp. nov.

*Pandoraea cepalis* sp. nov. [ce.pa’lis. Gr. n. *kepos*, garden; *-alis* L. adjective forming suffix, pertaining to; N.L. fem. adj. *cepalis* pertaining to garden (soil)].

The phenotypic description is as presented above and in Table 3.

Isolated from soil and water samples in Belgium and the Netherlands, from human clinical samples in the United States, and from historical bamboo slips in China.

The type strain is LMG 31106^T^ (=CCUG 39680^T^) and was isolated from garden soil in The Netherlands. Its G + C content is 63.7 mol% (calculated based on its genome sequence). The 16S rRNA, *gltB*, *gyrB*, *recA* and whole-genome sequence of LMG 31106^T^ are publicly available through the accession numbers LR536852, LR536896–LR536898, and CABPSL010000000, respectively.

### Description of *Pandoraea commovens* sp. nov.

*Pandoraea commovens* sp. nov. (com.mo’vens. L. v. *commovere*, to trouble, upset; L. pres. part. *commovens* troubling).

The phenotypic description is as presented above and in Table 3.

Isolated from human clinical samples in Belgium and the United States, from soil samples in Belgium, and from plant roots in India.

The type strain is LMG 31010^T^ (=CCUG 73378^T^) and was isolated from sputum of a cystic fibrosis patient in Belgium in 2002. Its G + C content is 62.6 mol% (calculated based on its genome sequence). The 16S rRNA, *gltB*, *gyrB*, *recA* and whole-genome sequence of LMG 31010^T^ are publicly available through the accession numbers LR536853, LR536902–LR536904, and CABPSA010000000, respectively.

### Description of *Pandoraea communis* sp. nov.

*Pandoraea communis* sp. nov. (com.mu’nis. L. fem. adj. *communis* common, widespread).

The phenotypic description is as presented above and in Table 3.

Isolated from human clinical, soil and water samples in Belgium, and from soil in Australia.

The type strain is LMG 31110^T^ (=CCUG 73383^T^) and was isolated from sputum of a cystic fibrosis patient in Belgium in 2012. Its G + C content is 62.6 mol% (calculated based on its genome sequence). The 16S rRNA, *gltB*, *gyrB*, *recA* and whole-genome sequence of LMG 31110^T^ are publicly available through the accession numbers LR536854, LR536911-LR536913 and CABPSJ010000000, respectively.

### Description of *Pandoraea eparura* sp. nov.

*Pandoraea eparura* sp. nov. (ep.a.ru’ra. Gr. masc. adj. *eparouros*, attached to the soil; N.L. fem. adj. *eparura* attached to the soil).

The phenotypic description is as presented above and in Table 3.

The type (and thus far only) strain is LMG 31012^T^ (=CCUG 73380^T^) and was isolated from soil of a house plant in Belgium in 2003. Its G + C content is 63.7 mol% (calculated based on its genome sequence). The 16S rRNA, *gltB*, *gyrB*, *recA* and whole-genome sequence of LMG 31012^T^ are publicly available through the accession numbers LR536855, LR536923–LR536925, and CABPSH010000000, respectively.

### Description of *Pandoraea horticolens* sp. nov.

*Pandoraea horticolens* sp. nov. (hor.ti’co.lens. L. n. *hortus* garden; L. v. *colere* to dwell; L. pres. part. *colens* dwelling; N.L. part. adj. *horticolens* because the type strain was isolated from garden [soil]).

The phenotypic description is as presented above and in Table 3.

The type (and thus far only) strain is LMG 31112^T^ (=CCUG 73379^T^) and was isolated from garden soil in Belgium in 2003. Its G + C content is 62.3 mol% (calculated based on its genome sequence). The 16S rRNA, *gltB*, *gyrB*, *recA* and whole-genome sequence of LMG 31112^T^ are publicly available through the accession numbers LR536857, LR536926-LR536928 and CABPSM010000000, respectively.

### Description of *Pandoraea iniqua* sp. nov.

*Pandoraea iniqua* sp. nov. (in.i’qua. L. fem. adj. *iniqua* disadvantageous, hostile).

The phenotypic description is as presented above and in Table 3.

Isolated from soil samples in Belgium and human clinical samples in the United States.

The type strain is LMG 31009^T^ (=CCUG 73377^T^) and was isolated from maize rhizosphere soil in Belgium in 2002. Its G + C content is 63.1 mol% (calculated based on its genome sequence). The 16S rRNA, *gltB*, *gyrB*, *recA* and whole-genome sequence of LMG 31009^T^ are publicly available through the accession numbers LR536856, LR536929–LR536931, and CABPSF010000000, respectively.

### Description of *Pandoraea morbifera* sp. nov.

*Pandoraea morbifera* sp. nov. (mor.bi’fe.ra, L. fem. adj. *morbifera* that brings disease).

The phenotypic description is as presented above and in Table 3.

Isolated from human clinical samples in the United States.

The type strain is LMG 31116^T^ (=CCUG 73389^T^) and was isolated from a cystic fibrosis specimen in the United States in 2006. Its G + C content is 64.7 mol% (calculated based on its genome sequence). The 16S rRNA, *gltB*, *gyrB*, *recA* and whole-genome sequence of LMG 31116^T^ are publicly available through the accession numbers LR536858, LR536935–LR536937, and CABPSD010000000, respectively.

### Description of *Pandoraea nosoerga* sp. nov.

*Pandoraea nosoerga* sp. nov. (no.so.er’ga, Gr. masc. adj. *nosoergos*, causing sickness; N.L. fem. adj. *nosoerga*).

The phenotypic description is as presented above and in Table 3.

Isolated from human clinical samples in Australia, Belgium, Germany, United Kingdom and the United States.

The type strain is LMG 31109^T^ (=CCUG 73390^T^) and was isolated from a cystic fibrosis specimen in the United States in 2008. Its G + C content is 66.1 mol% (calculated based on its genome sequence). The 16S rRNA, *gltB*, *gyrB*, *recA* and whole-genome sequence of LMG 31109^T^ are publicly available through the accession numbers LR536859, LR536941-LR536943 and CABPSC010000000, respectively.

### Description of *Pandoraea pneumonica* sp. nov.

*Pandoraea pneumonica* sp. nov. (pneu.mo’ni.ca, Gr. masc. adj. *pneumonikos*, of the lungs; N.L. fem. adj. *pneumonica*).

The phenotypic description is as presented above and in Table 3.

The type (and thus far only) strain is LMG 31114^T^ (=CCUG 73388^T^) and was isolated from a cystic fibrosis specimen in the United States in 2009. Its G + C content is 62.5 mol% (calculated based on its genome sequence). The 16S rRNA, *gltB*, *gyrB*, *recA* and whole-genome sequence of LMG 31114^T^ are publicly available through the accession numbers LR536861, LR536974–LR536976, and CABPSK010000000, respectively.

### Description of *Pandoraea soli* sp. nov.

*Pandoraea soli* sp. nov. (so’li. L. gen. n. *soli* of soil, the source of the type strain).

The phenotypic description is as presented above and in Table 3.

The type (and thus far only) strain is LMG 31014^T^ (=CCUG 73382^T^) and was isolated from soil of a house plant in Belgium in 2003. Its G + C content is 63.6 mol% (calculated based on its genome sequence). The 16S rRNA, *gltB*, *gyrB*, *recA* and whole-genome sequence of LMG 31014^T^ are publicly available through the accession numbers LR536860, LR536980–LR536982, and CABPSG010000000, respectively.

### Description of *Pandoraea terrigena* sp. nov.

*Pandoraea terrigena* sp. nov. (ter.ri’ge.na. L. fem. n. *terra* soil; L. v. *gignere* to bear; L. fem. n. *terrigena* [nominative in apposition] born of, or from, soil, soil-born).

The phenotypic description is as presented above and in Table 3.

The type (and thus far only) strain is LMG 31013^T^ (=CCUG 73381^T^) and was isolated from soil of a house plant in Belgium in 2003. Its G + C content is 63.5 mol% (calculated based on its genome sequence). The 16S rRNA, *gltB*, *gyrB*, *recA* and whole-genome sequence of LMG 31013^T^ are publicly available through the accession numbers LR536862, LR536977–LR536979, and CABPRU010000000, respectively.

## Data Availability Statement

The datasets generated for this study can be found in the European Nucleotide Archive PRJEB30806, PRJEB30685, PRJEB30686, PRJEB30687, PRJEB30688, PRJEB30689, PRJEB30690, PRJEB30807, PRJEB30745, PRJEB30746, PRJEB30808, PRJEB30691, PRJEB30692, PRJEB30809, PRJEB30810, PRJEB30693, PRJEB30694, PRJEB30695, PRJEB30696, PRJEB30697, PRJEB30699, PRJEB30700, PRJEB30701, PRJEB30698, PRJEB30811, PRJEB30702, PRJEB30703, PRJEB30812, PRJEB30704, PRJEB30705, PRJEB30706, PRJEB30707, PRJEB30708, PRJEB30714, PRJEB30709, PRJEB30710, PRJEB30711, PRJEB30712, PRJEB30713, PRJEB30813, PRJEB30814, PRJEB30815, PRJEB30755, PRJEB30724, PRJEB30756, PRJEB30725, PRJEB30726, PRJEB30727, PRJEB30728, PRJEB30721, PRJEB30722, PRJEB30723, PRJEB30757, PRJEB30715, PRJEB30716, PRJEB30717, PRJEB30753, PRJEB30752, PRJEB30754, PRJEB30740, PRJEB30741, PRJEB30742, PRJEB30743, PRJEB30718, PRJEB30744, PRJEB30748, PRJEB30749, PRJEB30750, PRJEB30751, PRJEB30729, PRJEB30730, PRJEB30731, PRJEB30732, PRJEB30733, PRJEB30734, PRJEB30735, PRJEB30736, PRJEB30737, PRJEB30738, PRJEB30739, PRJEB30747, PRJEB30720, and PRJEB30719.

## Author Contributions

PV, JL, and CP conceived the study. PV and CP wrote the manuscript. EDB, EDC, TS, and CP performed single locus sequence analyses. CP performed phylogenetic analyses. CP, ED, and BV carried out the genomic data analyses. EDC, MC, EDB, and CS carried out wet-lab phenotypical analyses. PV and JL generated the required funding. All authors read and approved the final manuscript.

## Conflict of Interest

The authors declare that the research was conducted in the absence of any commercial or financial relationships that could be construed as a potential conflict of interest.

## Footnotes

1 github.com/lh3/seqtk

2 http://ggdc.dsmz.de

## Abbreviations

ANI: average nucleotide identity
COG: cluster of orthologous groups
dDDH: digital DNA–DNA hybridization
GBDP: genome blast distance phylogeny
GGDC: genome-to-genome distance calculator
KEGG: kyoto encyclopedia of genes and genomes.
