# Supplementary material for: Comparative Genomics of Pandoraea, a Genus Enriched in Xenobiotic Biodegradation and Metabolism
Source: Front Microbiol. 2019 Nov 6;10:2556. doi: 10.3389/fmicb.2019.02556 (PMC6851202; doi:10.3389/fmicb.2019.02556)
Supplement: Supplementary file 1 [file Data_Sheet_1.PDF]

Supplementary Material

Tables

Table S1. Well-characterized reference strains and field isolates belonging to established *Pandoraea* species. CF, cystic fibrosis. LMG, BCCM/LMG Bacteria Collection, Laboratory of Microbiology, Ghent University, Ghent, Belgium; CCM, Czech Collection of Microorganisms, Masaryk University, Brno, Czech Republic; CCUG, Culture Collection University of Gothenburg, Department of Clinical Bacteriology, Sahlgrenska University Hospital, Gothenburg, Sweden; CDC, Centers for Disease Control, United States Public Health Service, Atlanta, USA; KACC, Korean Agricultural Culture Collection, National Institute of Agricultural Science and Technology, Suwon, Republic of Korea.

Strain	Other strains designations	Source	Depositor	Reference
Pandoraea apista				
LMG 16407^{T}	5893^{T}, CCUG 38412^{T}	CF sputum (Denmark)	N. Hoiby	(Coenye et al., 2000)
LMG 18089	CEP0633	CF sputum (United States)	D. P. Speert	(Coenye et al., 2000)
LMG 18818	AU0193	CF sputum (United States, 1997)	Own isolate	(Coenye et al., 2000)
R-10112	AU1465	CF sputum (United States, 1999)	Own isolate	
R-14454	AU2513	CF sputum (United States, 2000)	Own isolate	
R-15345	AU2160	CF sputum (United States, 2000)	Own isolate	
R-31796	54459A101	CF sputum (Denmark)	N. Hoiby	
Pandoraea faecigallinarum				
LMG 28171^{T}	CCM 2766^{T}	Chicken, dung	CCM	(Sahin et al., 2011)
Pandoraea fibrosis				
LMG 29626^{T}	6399^{T}	CF sputum (Australia, 2012)	R. Ee	(See-Too et al., 2019)
LMG 31113	AU17150	CF patient (United States, 2008)	Own isolate	
R-11943	CUL 264	Water (Lebanon, 1996)	J.-M. Meyer	
Pandoraea norimbergensis				
LMG 18379^{T}	CCUG 39188^{T}	Water (Germany)	CCUG	(Coenye et al., 2000)
LMG 13019	90/072	Human blood (Belgium, 1990)	S. Lauwers	(Coenye et al., 2000)
LMG 16603	CCUG 34867	Human bronchial alveolar lavage (Sweden, 1995)	CCUG	(Coenye et al., 2000)

Pandoraea oxalativorans				
LMG 28169^T	CCM7677^T	Rhizosphere soil (Turkey)	CCM	(Sahin et al., 2011)
Pandoraea pnomenusa				
LMG 18087^T	C1513^T, CCUG 38742^T	CF sputum (United Kingdom)	J. R. W. Govan	(Coenye et al., 2000)
LMG 18817	PC13	CF patient (United States, 1986)	Own isolate	(Coenye et al., 2000)
LMG 18820	PHA 1119	Sludge (Germany)	J. Mergaert	(Coenye et al., 2000)
LMG 24771	AI-S130	Plant (Azadirachta indica A. Juss) rhizoplane (India)	M. Madhaiyan	
LMG 31119	AU1039	CF patient (United States, 1999)	Own isolate	
R-1454	C7351	CF sputum (Canada)	D. P. Speert	(Coenye et al., 2000)
R-4805	HI2344	CF patient (United States, 1998)	Own isolate	(Coenye et al., 2000)
R-7719	K160189KP	CF patient (Belgium, 1999)	H. Franckx	
R-7902	K240586BA	CF patient (Belgium, 1999)	H. Franckx	
R-15667	CDC G8107	Human Blood (United States, 1993)	CDC	(Daneshvar et al., 2001)
Pandoraea pulmonicola				
LMG 18106^T	FC330^T, CCUG 38759^T	CF patient (Canada)	D. P. Speert	(Coenye et al., 2000)
LMG 18107	L. Saimann41	CF patient (United States)	L. Saimann	(Coenye et al., 2000)
LMG 18108	L. Saimann42	CF patient (United States)	L. Saimann	(Coenye et al., 2000)
Pandoraea sputorum				
LMG 18819^T	AU0124^T, CCUG 39682^T	CF patient (United States, 1997)	Own isolate	(Coenye et al., 2000)
LMG 18100	C4946	CF patient (Canada)	D. P. Speert	(Coenye et al., 2000)
LMG 18109	L. Saimann57, HI2111	CF patient (United States)	L. Saimann	
LMG 20601	CDC G5084	Human sinus (United States, 1990)	CDC	(Daneshvar et al., 2001)
LMG 31120	AU1359	CF patient (United States, 1999)	Own isolate	
LMG 31121	V02 34922	CF patient (Switzerland, 2007)	R. Zbinden	
R-2456	PC212	Source unknown (United States, 1987)	Own isolate	(Coenye et al., 2000)
R-11656	AU1831	CF patient (United States, 2000)	Own isolate	
R-13191	AU2075	CF patient (United States, 2000)	Own isolate	
R-13758	AU2302	CF patient (United States, 2000)	Own isolate	

R-13761	AU2321	CF patient (United States, 2000)	Own isolate	
R-14315	AU2389	CF patient (United States, 2000)	Own isolate	
Pandoraea terrae				
LMG 30175^T	SE-S21^T, JCM 30137^T	Forest soil (South Korea, 2014)	JCM	(Jeong et al., 2016)
Pandoraea thiooxydans				
LMG 24779^T	KACC 12757^T	Rhizosphere soil (Sesamum indicum L.) (South Korea)	KACC	(Anandham et al., 2010)
Pandoraea vervacti				
LMG 28170^T	CCM 7667^T, NS15^T	Soil (Turkey)	CCM	(Sahin et al., 2011)

Table S2. Genomes sequenced in the present study.

Strain	Project	Contigs	Size (bp)	N50 (bp)	Coverage (x)	%GC	CDS
P. apista LMG 18089	PRJEB30685	27	5,815,466	457,211	134	62.7	5,279
P. fibrosis LMG 31113	PRJEB30745	43	5,605,513	483,367	185	62.8	4,943
P. pnomenusa LMG 31119	PRJEB30696	46	5,305,298	262,716	119	64.9	4,699
P. sputorum LMG 20601	PRJEB30706	62	6,264,179	471,825	155	62.7	5,539
P. sputorum LMG 31120	PRJEB30707	17	5,956,418	751,203	114	62.7	5,301
P. sputorum LMG 31121	PRJEB30708	113	6,453,978	322,419	117	62.8	5,652
P. terrae LMG 30175^T	PRJEB30813	81	6,176,823	194,136	111	62.8	5,575
P. anapnoica sp. nov. LMG 31117^T	PRJEB30755	48	6,126,688	278,466	141	62.4	5,364
P. anhela sp. nov. LMG 31108^T	PRJEB30724	61	6,046,012	256,277	138	63.4	5,188
P. aquatica sp. nov. LMG 31011^T	PRJEB30756	17	5,958,127	792,622	123	62.9	5,238
P. bronchicola sp. nov. LMG 20603^T	PRJEB30725	34	5,351,123	323,330	158	63.0	4,753
P. capi sp. nov. LMG 20602^T	PRJEB30721	31	5,852,144	401,082	115	63.4	5,056
P. captiosa sp. nov. LMG 31118^T	PRJEB30757	36	6,139,582	614,074	134	63.3	5,340
P. cepalis sp. nov. LMG 31106^T	PRJEB30715	56	5,274,229	187,061	208	63.7	4,730
P. cepalis sp. nov. LMG 31107	PRJEB30716	32	5,159,566	300,362	131	63.5	4,626
P. commovens sp. nov. LMG 31010^T	PRJEB30753	26	6,036,949	540,061	122	62.6	5,308
P. communis sp. nov. LMG 31110^T	PRJEB30740	17	5,708,603	837,780	139	62.6	5,067
P. communis sp. nov. LMG 31111	PRJEB30741	55	5,566,071	278,275	150	62.5	5,064
P. eparura sp. nov. LMG 31012^T	PRJEB30718	35	5,205,577	435,937	135	63.7	4,621
P. horticolens sp. nov. LMG 31112^T	PRJEB30744	68	6,008,490	290,798	122	62.3	5,378
P. iniqua sp. nov. LMG 31009^T	PRJEB30748	17	6,339,129	1,113,537	117	63.1	5,521
P. iniqua sp. nov. LMG 31115	PRJEB30749	14	6,296,634	1,159,992	143	63.1	5,445
P. morbifera sp. nov. LMG 31116^T	PRJEB30750	47	5,233,298	316,192	161	64.7	4,676
P. nosoerga sp. nov. LMG 31109^T	PRJEB30729	41	4,862,114	229,370	205	66.1	4,266
P. pneumonica sp. nov. LMG 31114^T	PRJEB30747	12	5,845,078	1,392,766	181	62.5	5,202
P. soli sp. nov. LMG 31014^T	PRJEB30720	51	4,961,982	370,563	145	63.6	4,395

P. terrigena sp. nov. LMG 31013^T	PRJEB30719	35	5,356,606	374,338	142	63.5	4,878

Table S7. The frequency of orthologous versus non-orthologous CDS varies among species.

Pearson chi-square analysis testing the independence of gene conservation (orthologous vs. non-orthologous CDS) and species ($X^2(29) = 5863$, $p<0.001$). Each cell in the contingency represents the observed frequency and standardized residual (in between brackets) and is preceded by + or - if the standardized residual is >1.96 or <-1.96, respectively, and significant at $p<0.05$.

		Orthologous CDS		Non-orthologous CDS
Ca. P. novymonadis		920 (-1.058)	+	48 (8.344)
P. anapnoica		5307 (0.385)	-	57 (-3.032)
P. anhela		5012 (-1.313)	+	176 (10.352)
P. apista	+	97304 (4.003)	-	305 (-31.560)
P. aquatica		5118 (-0.516)	+	120 (4.067)
P. bronchicola		4649 (-0.420)	+	104 (3.311)
P. capi		10134 (-1.117)	+	278 (8.808)
P. captiosa		5221 (-0.475)	+	119 (3.744)
P. cepalis		13600 (-0.901)	+	326 (7.101)
P. commovens		5226 (0.029)		82 (-0.225)
P. communis		15005 (-0.261)	+	274 (2.059)
P. eparura		4510 (-0.561)	+	111 (4.421)
P. faecigallinarum		4719 (-0.898)	+	139 (7.076)
P. fibrosis		13584 (1.193)	-	78 (-9.407)
P. horticolens		5168 (-1.716)	+	210 (13.526)
P. iniqua		10821 (0.276)	-	145 (-2.175)
P. morbifera		4582 (-0.294)	+	94 (2.318)
P. norimbergensis		5177 (0.320)	-	60 (-2.519)
P. nosoerga		4188 (-0.161)		78 (1.270)
P. oxalativorans		5237 (-1.063)	+	163 (8.378)
P. pneumonica		5096 (-0.330)	+	106 (2.602)
P. pnomenusa		32135 (0.920)	-	350 (-7.251)
P. pulmonicola		4719 (-1.039)	+	149 (8.189)
P. soli		4346 (0.313)	-	49 (-2.470)
P. sputorum		21040 (0.017)		336 (-0.138)
P. terrae	-	4943 (-7.340)	+	632 (57.864)
P. terrigena		4758 (-0.617)	+	120 (4.863)
P. thiooxydans	-	8010 (-2.688)	+	377 (21.187)
P. vervacti		4758 (0.337)	-	53 (-2.657)
Pandoraea sp.		10592 (0.628)	-	105 (-4.949)

Table S8. Orthogroup specificity varies among COG categories.

Pearson chi-square analysis testing the independence of orthogroup specificity and COG category ($X^2(66) = 522$, $p<0.001$). Each cell in the contingency represents the observed frequency and standardized residual (in between brackets) and is preceded by + or - if the standardized residual is >1.96 or <-1.96, respectively, and significant at $p<0.05$.

			Core		Multiple species		Single species		Single isolate
**Information storage and processing**									
J	Translation, ribosomal structure and biogenesis	+	159 (11.999)	-	114 (-6.199)		12 (-1.625)		0 (-0.573)
K	Transcription	-	97 (-5.399)	+	598 (2.897)		54 (0.535)		0 (-0.929)
L	Replication, recombination and repair	+	63 (2.042)		144 (-0.909)		12 (-0.699)		0 (-0.503)
B	Chromatin structure and dynamics		1 (0.116)		2 (-0.497)		1 (1.413)		0 (-0.068)
**Cellular processes and signaling**									
D	Cell cycle control, cell division, chromosome partitioning	+	26 (3.032)		39 (-1.046)	-	0 (-2.087)		0 (-0.274)
V	Defense mechanisms	-	22 (-2.371)	+	137 (1.990)	-	4 (-2.095)		0 (-0.434)
T	Signal transduction mechanisms		67 (-0.346)		226 (0.221)		21 (-0.010)		0 (-0.602)
M	Cell wall/membrane/envelope biogenesis		105 (0.214)		322 (-0.313)		34 (0.545)		1 (0.640)
N	Cell motility		43 (-0.882)		166 (0.739)		12 (-0.731)		0 (-0.505)
W	Extracellular structures		0 (-1.334)		8 (0.977)		0 (-0.732)		0 (-0.096)
U	Intracellular trafficking, secretion, and vesicular transport		26 (-0.738)		107 (1.150)	-	2 (-2.343)		0 (-0.395)
O	Posttranslational modification, protein turnover, chaperones	+	85 (4.521)	-	130 (-2.841)		20 (1.070)		0 (-0.521)
**Metabolism**									
C	Energy production and conversion	+	125 (2.065)		301 (-1.660)		41 (1.733)		0 (-0.734)
G	Carbohydrate transport and metabolism	-	63 (-3.095)		318 (1.294)		36 (1.522)		0 (-0.693)
E	Amino acid transport and metabolism		168 (0.594)		498 (-0.591)		54 (0.816)		1 (0.185)
F	Nucleotide transport and metabolism	+	55 (5.409)	-	54 (-3.435)		12 (1.366)		0 (-0.374)
H	Coenzyme transport and metabolism	+	96 (3.950)		181 (-1.674)		12 (-1.675)		0 (-0.577)
I	Lipid transport and metabolism		99 (-0.005)		322 (0.360)		24 (-1.067)		0 (-0.716)
P	Inorganic ion transport and metabolism	-	67 (-2.579)		315 (1.333)		30 (0.453)		0 (-0.689)
Q	Secondary metabolites biosynthesis, transport and catabolism		29 (-1.175)		122 (0.663)		10 (-0.261)		1 (1.881)
**Poorly characterized**									
R	General function prediction only	-	81 (-2.113)		341 (0.817)		37 (1.110)		1 (0.645)
S	Function unknown	-	66 (-2.724)		315 (1.247)		32 (0.807)		1 (0.756)
**Mobile elements**									
X	Mobilome: prophages, transposons	-	1 (-5.970)	+	160 (3.666)		5 (-1.880)	+	3 (6.354)

Table S9. Orthogroup specificity varies among KEGG categories.

Pearson chi-square analysis testing the independence of orthogroup specificity and KEGG category ($X^2(10) = 130$, $p<0.001$). Each cell in the contingency represents the observed frequency and standardized residual (in between brackets) and is preceded by + or - if the standardized residual is >1.96 or <-1.96, respectively, and significant at $p<0.05$.

		Core		Multiple species	Single species
09100 Metabolism		511 (-0.192)		729 (0.118)	51 (0.166)
09120 Genetic Information Processing	+	136 (7.432)	-	41 (-6.062)	5 (-0.764)
09130 Environmental Information Processing	-	83 (-4.160)	+	227 (3.183)	17 (1.232)
09140 Cellular Processes		44 (-1.044)		84 (1.347)	1 (-1.783)
09150 Organismal Systems		3 (0.123)		4 (0.033)	0 (-0.520)
09160 Human Diseases		9 (-1.150)		22 (0.800)	2 (0.644)

Figure legends

Figure S1. Phylogenetic tree based on partial *recA* sequences of all *Pandoraea* strains examined. Sequences (621-663 bp) were aligned based on their amino acid sequences and phylogeny was inferred using the Maximum Likelihood method and GTRCAT substitution model in RAxML. The percentage of replicate trees in which the associated taxa clustered together in the bootstrap test (1000 replicates) are shown next to the branches if greater than 50%. *Burkholderia cenocepacia* J2315^{T} was used as outgroup. The scale bar indicates the number of substitutions per site. Isolates selected for whole-genome sequencing are shown in bold character type.

Figure S2. Phylogenetic tree based on partial *gltB* sequences of all *Pandoraea* strains examined. Sequences (600-609 bp) were aligned based on their amino acid sequences and phylogeny was inferred using the Maximum Likelihood method and GTRCAT substitution model in RAxML. The percentage of replicate trees in which the associated taxa clustered together in the bootstrap test (1000 replicates) are shown next to the branches if greater than 50%. *Burkholderia cenocepacia* J2315^{T} was used as outgroup. The scale bar indicates the number of substitutions per site. Isolates selected for whole-genome sequencing are shown in bold character type.
